# Gut microbial dysbiosis after traumatic brain injury modulates the immune response and impairs neurogenesis

**DOI:** 10.1186/s40478-021-01137-2

**Published:** 2021-03-10

**Authors:** Marta Celorrio, Miguel A. Abellanas, James Rhodes, Victoria Goodwin, Jennie Moritz, Sangeetha Vadivelu, Leran Wang, Rachel Rodgers, Sophia Xiao, Ilakkia Anabayan, Camryn Payne, Alexandra M. Perry, Megan T. Baldridge, Maria S. Aymerich, Ashley Steed, Stuart H. Friess

**Affiliations:** 1 Division of Critical Care Medicine, Department of Pediatrics, Washington University in St. Louis School of Medicine, St. Louis, USA; 2 Division of Infectious Diseases, Department of Medicine, Washington University in St. Louis School of Medicine, St. Louis, USA; 3 Departamento de Bioquímica Y Genética, Facultad de Ciencias, Universidad de Navarra, Pamplona, Spain; 4 CIMA, Programa de Neurociencias, Universidad de Navarra, Pamplona, Spain; 5 IdiSNA, Instituto de Investigación Sanitaria de Navarra, Pamplona, Spain

**Keywords:** Traumatic brain injury, Gut microbial dysbiosis, Antibiotics, Fear conditioning, Microglia, Monocytes, T cells, Neurogenesis

## Abstract

The influence of the gut microbiota on traumatic brain injury (TBI) is presently unknown. This knowledge gap is of paramount clinical significance as TBI patients are highly susceptible to alterations in the gut microbiota by antibiotic exposure. Antibiotic-induced gut microbial dysbiosis established prior to TBI significantly worsened neuronal loss and reduced microglia activation in the injured hippocampus with concomitant changes in fear memory response. Importantly, antibiotic exposure for 1 week after TBI reduced cortical infiltration of Ly6C^high^ monocytes, increased microglial pro-inflammatory markers, and decreased T lymphocyte infiltration, which persisted through 1 month post-injury. Moreover, microbial dysbiosis was associated with reduced neurogenesis in the dentate gyrus 1 week after TBI. By 3 months after injury (11 weeks after discontinuation of the antibiotics), we observed increased microglial proliferation, increased hippocampal neuronal loss, and modulation of fear memory response. These data demonstrate that antibiotic-induced gut microbial dysbiosis after TBI impacts neuroinflammation, neurogenesis, and fear memory and implicate gut microbial modulation as a potential therapeutic intervention for TBI.

**Supplementary Information:**

The online version contains supplementary material available at 10.1186/s40478-021-01137-2.

## Introduction

Despite advances in care, morbidity and mortality after traumatic brain injury (TBI) remain high [20]. In 2013, a total of approximately 2.8 million TBI visits, 282,000 TBI-related hospitalizations, and 56,000 TBI-related deaths occurred in the United States [70]. Following the primary injury, secondary injuries develop from multiple mechanisms including inflammation, hypoxemia, ischemia, cerebral edema, and excitotoxicity. Indeed, TBI is characterized as a complex neurological insult that results from initial brain trauma and can lead to chronic, progressive deterioration of patient health (impaired cognition, fatigue, and impaired socialization) [5, 67]. However, aside from these deleterious effects, posttraumatic inflammation mediates neuroreparative mechanisms after TBI [62]. Phenotypic changes in microglial/macrophage activation can influence multiple steps in central nervous system (CNS) repair and regeneration through neurogenesis, angiogenesis, oligodendrogenesis, and remyelination [44].

Adult neurogenesis in the hippocampus has been demonstrated in rodents [6, 52, 78] primates, and humans [77] to be influenced by both local and systemic insults. Gut microbiome alteration has been associated with diminishing hippocampal neurogenesis and memory retention that have been rescued by cognitive activity such as learning, environmental enrichment [78], probiotics, and exercise [52]. In addition, an increase of progenitor cells and enhanced neurogenesis have been reported after TBI [73]. Recruitment of T cells and activation of microglia in the brain play important roles in the maintenance of hippocampus neurogenesis and memory [78]. T cells and microglia interactions in the brain could mediate brain plasticity and cell renewal in the hippocampus.

The impact of the host microbiota on the inflammatory and repair response after TBI is unknown. Remarkably, a recent study has described the gut microbiota as being altered in chronic, moderate-severe TBI patients in permanent care facilities [48]. A body of literature related to the gut microbiota’s influence on the brain and behavior has emerged [33]. The gut microbiota has been implicated in important and, surprisingly, distant roles beyond the promotion of nutrient digestion and intestinal health [33]. For instance, microbial dysbiosis has been linked to alterations in blood brain barrier (BBB) permeability and microglial activation [7, 26]. Likewise, commensal gut bacteria are instrumental for immune system development, maintenance, and function [4, 15, 33, 72]. Important interactions between the microbiota and the CNS have been highlighted by recent studies performed in ischemic stroke models [4, 66] and a broad-spectrum antibiotic-treated mouse model [52]. These studies suggest that changes in gut microbiota can alter the peripheral immune cell response to primary brain injury [66]. Strikingly, the gut-brain interaction is bidirectional as TBI can also alter the composition of microbial communities [71].

The gut microbiota’s influence on TBI is of paramount clinical significance as TBI patients are highly susceptible to alterations in the gut microbiota due to frequent antibiotic administration, prolonged hospitalization, and autonomic dysfunction. Characterization of local and peripheral immune cells which reside in or infiltrate the brain, respectively and their functional roles in brain homeostasis is important to investigate how gut microbial dysbiosis contributes to neuronal vulnerability after injury. Microglial activity can be influenced by factors originating outside the CNS including the gut [15] as well as the peripheral immune system [61]. We hypothesize that microbial dysbiosis leads to worsened outcomes after TBI, and this effect is mediated by microbial-host immune interactions, which are dependent on changes induced by the peripheral and/or local immunological environment.

In this current study, microbial dysbiosis via broad-spectrum antibiotic administration before, during, and after TBI induced a significant change in gut microbiota, increased neuronal loss, suppressed post-injury hippocampal neurogenesis, altered the microglia and peripheral immune response, and was associated with long-term contextual memory alteration. These findings point to the important role of the gut microbiota during recovery from TBI and implicate microbial modulation of the ensuing immune response as its protective mechanism.

## Materials and methods

### TBI murine model with induction of microbial dysbiosis

TBI murine in vivo study with induction of microbial dysbiosis was utilized to elucidate the influence that gut microbiota has on TBI, tissue repair, and subsequent neurological outcomes. All procedures were approved by the Washington University Animal Studies Committee (Protocol 20190223) and are consistent with the National Institutes of Health guidelines for the care and use of animals. Animals were housed 5/cage and had free access to water and food with 12 h (h) light/dark cycle. C57BL/6 J 6–8 week old male mice (Jackson Laboratory, Bar Harbor, ME) weighing 20–25 g (g) were used. Briefly, mice were anesthetized with 5% isoflurane at induction, followed by maintenance at 2% isoflurane for the procedure’s duration. Buprenorphine sustained release (0.5 mg/kg subcutaneously) was administered before scalp incision. The head was shaved and ear bars were used to stabilize the head within the stereotaxic frame (MyNeurolab, St. Louis, MO). Then, a single 5-mm craniectomy was performed by an electric drill on the left lateral side of the skull centered 2.7 mm lateral from the midline and 3 mm anterior to lambda. Animals were randomized to sham or injury after craniectomy using computer-generated number randomization. For injured animals, the 3-mm electromagnetic impactor tip was then aligned with the craniectomy site at 1.2 mm left of midline and 1.5 mm anterior to the lambda suture. The impact was delivered at 2 mm depth (velocity 5 m/s, dwell time 100 ms). The ears bars were released immediately after the injury. All animals then received a loose fitting plastic cap secured over the craniectomy with Vetbond (3 M, St. Paul, MN). The skin was closed with interrupted sutures and was treated with antibiotic ointment before the mouse was recovered from anesthesia on a warming pad. To induce microbial dysbiosis we used broad-spectrum antibiotics in the drinking water: 250 mg vancomycin, 500 mg neomycin-sulfate, 500 mg ampicillin, 500 mg metronidazole (VNAM), and 10 g grape Kool-Aid (Kraft Heinz, IL, Chicago) in 500 mL water which was sterile filtered through a 0.22 µm filter [68]. Control groups of sham and injured animals received Kool-Aid alone in the drinking water for the same duration as the experimental groups. All animals surviving longer than 1 week resumed regular drinking water 7 days after surgery. Mice were survived for 3 days, 1 week, 1 month, or 3 months after injury.

### Fecal bacterial quantification

Fecal pellets were collected into sterile 1.7 ml tubes. Samples were homogenized in mpbio Lysing Matrix D (1.4 mm ceramic spheres) tubes in PBS for 5 min at maximum speed using a Qiagen TissueLyser LT, and DNA was isolated from the aqueous phase using QIAamp DNA Stool Mini Kit (QIAGEN Inc., Valencia, CA) per the manufacturer’s instructions. Purified DNA was subject to sequencing at the McDonnell Genome Institute at Washington University using the Fluidigm Access Array System to generate 7 PCR amplicons, representing all nine 16S variable regions. The sample inlets consisted of 1X High Fidelity FastStart Reaction Buffer without MgCl2 (Roche, Basel, Switzerland), 4.5 nM MgCl2 (Roche), 5% DMSO (Roche), 200 uM PCR Grade Nucleotide Mix (Roche), 0.05 U/μL 5 U/μL FastStart High Fidelity Enzyme Blend (Roche), 1X Access Array Loading Reagent (Fluidigm), and 5 ng DNA. The forward and reverse primers as indicated in Table 1 were added to the assay inlets at 200 nM with 1X Access Array Loading Reagent. The samples were harvested after PCR amplification was performed on the BioMark HD system from Fluidigm and indexed using unique 10 base pair sequences with 14 rounds of PCR to incorporate each index sequence. All samples were pooled into 48 sample libraries and cleaned using bead purification. The samples were loaded on Miseq instruments and sequenced using the 2 × 250 bp protocol. Fastq sequencing data files were demultiplexed using the MVRSION pipeline. Read quality control and the resolution of amplicon sequence variants with the forward-reads were performed using the DADA2 R package (version 1.14.1). Taxonomy was assigned using the DADA2-formatted training files derived from the Ribosomal Database Project’s Training Set 16. Amplicon sequence variants were aligned and arranged into a maximum likelihood phylogeny using the Phangorn package. PhyloSeq objects were derived from the amplicon sequence variant counts, taxonomy, and phylogeny assignments using the Phyloseq package and then subject to diversity analysis.

**Table 1:** Primers sequences used to generate 7 PCR amplicons covering the variable regions in the bacterial 16S rRNA gene

Gene	Forward primer	Reverse primer
V1-V2	5′-TCGTCGGCAGCGTCAGAGTTTGATCCTGGCTCAG-3’	5′-GTCTCGTGGGCTCGGTGCTGCCTCCCGTAGGAGT-3’
V2	5′-TCGTCGGCAGCGTCAGYGGCGIACGGGTGAGTAA-3’	5′-GTCTCGTGGGCTCGGCYIACTGCTGCCTCCCGTAG-3’
V3_2	5′-TCGTCGGCAGCGTCCCTACGGGAGGCAGCAG-3’	5′-GTCTCGTGGGCTCGGGTATTACCGCGGCTGCTGG-3’
V4	5′-TCGTCGGCAGCGTCGTGCCAGCMGCCGCGGTAA-3’	5′-GTCTCGTGGGCTCGGGGACTACHVGGGTWTCTAAT-3’
V5-V6	5′-TCGTCGGCAGCGTCAGGATTAGATACCCTGGTA-3’	5′-GTCTCGTGGGCTCGGCRRCACGAGCTGACGAC-3’
V6_1	5′-TCGTCGGCAGCGTCAAACTCAAAKGAATTGACGG-3’	5′-GTCTCGTGGGCTCGGACGAGCTGACGACARCCATG-3’
V7-V8	5′-TCGTCGGCAGCGTCGYAACGAGCGCAACCC-3’	5′-GTCTCGTGGGCTCGGGACGGGCGGTGWGTRC-3’

### Novel object recognition test

TBI in humans frequently leads to cognitive dysfunction [42]. The novel object recognition test is a commonly used behavioral test to measure cognitive dysfunction. The present protocol is a modified version which was previously published [41]. This test allows measurements of memory and learning, the preference for novelty, and the influence that different brain regions have in the process of recognition. Mice were handled by the experimenter twice a day 2 days before the beginning of the behavioral test. For the performance of the test, we used a square 4-chamber open field apparatus made of grey durable Plexiglas material (40 × 40 × 30 cm), which had the advantage of being non-absorbent to odors and easy to clean. The floor was divided into 4 equal arenas for the direct counting of animal activity of 4 mice simultaneously. The luminosity of the room was adjusted with a luxmeter to obtain a light intensity of 20 lx in the center of the open field box, and the animal activity was recorded automatically by a SMART video tracking software (Panlab Harvard apparatus, Barcelona, Spain) using an overhead USB-camera (Logitech, Newark, CA). On day 1 and 2 (habituation phase), mice were placed in the different arenas and allowed to explore the space for 5 min (min) per day. On day 3 (familiarization session), mice were placed in the open field box in contact with two identical objects (towers of Lego bricks) at 5 cm from the walls for 10 min. On day 4 (test day), mice were returned to the arenas where one of the objects was changed for a new object (small falcon tissue culture flask half-filled with mouse bedding). On day 1 and 2, time in center, time in periphery, and total distance were analyzed. We calculated the Discrimination Index (DI), allowing discrimination between the novel and familiar objects, i.e., the exploration time for novel object (TN) was divided by the total amount of time interacting with the novel and familiar objects (TF): %DI = (100 × TN)/(TN + TF). After every session, the open field box and objects were cleaned with 70% ethanol to minimize olfactory cues.

### Contextual and cued fear conditioning

To study fear response in mice we used a modified version of previously published protocols of Pavlovian fear conditioning [39]. Fear conditioning tests are a type of associative learning task in which mice learn to associate a particular neutral conditioned stimulus (CS), a 5 kHz 70 dB tone, with an aversive unconditioned stimulus (US), a mild electrical foot-shock, and show a conditioned response (CR), freezing. After 5 repeated pairings of CS and US, the animal is conditioned to fear both the tone and training context. Two different contexts, context A and B, were used in fear conditioning. Mice were handled by the experimenter twice a day 2 days before the beginning of the behavioral test. For the performance of the test, we used a fear conditioning-system (Ugo Basile, Gemonio, Italy) consisting of a sound-attenuating box (48.5 × 38.5 × 48.5 cm) with ventilating fan, a light, overhead USB-camera (Logitech), and an electrical grid floor for inducing the foot-shocks. On day 1 (conditioning), mice were placed in context A and every 1.5 min they received 5 tone-shock pairings( 30 s tone with 0.5 mA and shock during the last 2 s) and the freezing time in 30 s epochs was measured by a blinded observer. On day 2 (contextual test), mice were placed in context A for 10.5 min and freezing time was measured to assess contextual fear memory. On day 3 (cued test), mice were placed in a novel context B (checkered walls and white hard cover on the floor) to eliminate any confounding interactions of contextual fear for 10.5 min and subsequently given five 30 s tones without any shocks. On day 1 and 3, after the final tone-shock pairing, mice remained in the conditioning chamber for 30 s before being returned to their home cages. Freezing was defined as the absence of visible movement except that required for respiration. Percentage of total freezing time was calculated by dividing the amount of time-spent freezing by total time (630 s).

### Tissue processing

Mice were anesthetized by isoflurane followed by transcardial perfusion with ice-cold 0.1 M heparinized phosphate-buffered saline (PBS, pH 7.4) followed by 4% paraformaldehyde (PFA, Sigma-Aldrich, St Louis, MO). Perfused brains were kept in 4% PFA at 4 ˚C overnight, followed by equilibration in 30% sucrose for at least 48 h before sectioning. For biochemical techniques, brains regions were rapidly removed, dissected, and flash frozen.

### Cell suspensions

The blood and the regions of interest in the ipsilateral side of the brain (hippocampus, amygdala, and cortex) were taken. Five animals (all the conditions) were processed per day with a total of 20 samples at the time. Mice were anesthetized with isoflurane, and blood samples were taken in EDTA tubes immediately before transcardial perfusion with ice-cold 0.1 M heparinized-PBS. The brain regions of interest were dissected out on ice and digested at 37 °C for 15 min with collagenase D (400 units/mL, Roche) in Dulbecco’s PBS (Lonza, Basel, Switzerland), each containing 50 μg/mL of DNase I (Sigma-Aldrich). The tissue was then mechanically dissociated with a glass Pasteur pipette, filtered through a 70-μm nylon cell strainer, and centrifuged at 950 rpm for 15 min. A 25% Percoll (Sigma-Aldrich) column was used to remove cell debris and myelin, followed by centrifugation at 1700 rpm for 10 min. 50 µl-blood sample was mixed with 1 × Red Blood Lysis Buffer (Roche) and incubated in rotation for 15 min at room temperature (RT). Samples were then centrifuged at 3500 rpm for 5 min at RT. The supernatant was discarded, and cells were washed and resuspended in 1 mL of cytometer buffer [0.5% bovine serum albumin (Sigma-Aldrich), 5 mM EDTA (Millipore, Burlington, MA) in PBS]. The cells were resuspended in 100 µl of cytometer buffer and stained.

### Flow cytometry analysis

Cells were incubated for 5 min at RT with Zombie NIR Dye (BioLegend, San Diego, CA, USA) to assess their viability. The Zombie NIR Dye was quenched, and cells were washed with cytometry buffer and blocked with FcR blocking reagent (1:50, Miltenyi Biotec, Bergisch Gladbach, Germany). Then, the samples were washed with cytometry buffer, stained with antibodies (Table 2) for 15 min at RT, and analyzed on a BD LSRFortessa flow cytometer (BD Biosciences, Franklin Lakes, NJ) using the Software v10.6.1 (BD Biosciences, Franklin Lakes, NJ). Microglial cells were defined as CD45^low^CD11b^+^ and T cells as CD45^high^CD11b^−^CD3^+^. Fluorescence minus one (FMO) and isotype control antibodies were used as negative controls for each marker.

**Table 2:** Overview of the primary antibodies used in the present study

Antibody	Fluorophore	Clone	Species	Dilution		Source	Product number
				Tissue	In vitro		
				IHQ	FC		
CD45	BV425	30-F11	Rat monoclonal		1:200	BioLegend	103,134
CD3ɛ	AF700	500-A2	Armenian Hamster monoclonal		1:100	BioLegend	100,320
CD4	BUV395	GK1.5	Rat monoclonal		1:250	BD Biosciences	565,974
CD8a	PerCP-Cy5.5	53–6.7	Rat monoclonal		1:200	BioLegend	100,733
CD11b	BV510	M1/70	Rat monoclonal		1:500	BioLegend	101,263
CD25	PE	PC61	Rat monoclonal		1:200	BioLegend	102,007
MHC-II	PerCP-710	AF6-120.1	Mouse monoclonal		1:200	eBioscience	46–5320-80
TLR4	PE-Cy7	SA15-21	Rat monoclonal		1:250	BioLegend	145,407
Ly6C	BV785	HK1.4	Rat monoclonal		1:2000	BioLegend	128,041
Ly6G	AF700	1A8	Rat monoclonal		1:100	BioLegend	127,622
NeuN		A60	Mouse monoclonal	1:1000		Millipore	MAB377
NeuN		A60	Rabbit polyclonal	1:4000		Millipore	MAB377
DCX			Rabbit polyclonal	1:1000		Abcam	Ab18723
BrdU		BU1/75	Rat monoclonal	1:150		Abcam	Ab6326
Iba1		NCNP24	Rabbit polyclonal	1:1000		Wako	019–19,741
CD3		SP7	Rabbit polyclonal	1:100		Abcam	ab16669
Secondary antibody	AF594		Donkey anti-rat	1:500		Thermo Fisher	A-21209
Secondary antibody	AF647		Donkey anti-mouse	1:500		Thermo Fisher	A-31571
Secondary antibody	AF488		Donkey anti-rabbit	1:500		Thermo Fisher	A-21206
Secondary antibody			Biotinylated goat anti-rabbit	1:1000		Vector Laboratories	BA-1000–1.5

### BrdU treatment

For the analysis of neurogenesis, the animals received intraperitoneal injections of 5-bromodeoxyuridine (BrdU, Sigma-Aldrich) 50 mg/g of body weight at a concentration of 10 mg/ml in sterile saline for 4 consecutive days.

### Immunohistochemistry

Histological sections were examined to assess pathological changes in the hippocampus. Immunohistochemistry was performed on free-floating sections (50-µm thick). The serial sections were cut on a freezing microtome starting with the appearance of a complete corpus callosum and caudally to bregma − 3.08 mm. Sets of 12 sections spaced every 300 µm were mounted on glass slides and used for immunohistochemical studies. Staining was performed on free-floating sections washed in Tris-buffered saline (TBS) between applications of primary and secondary antibodies. Endogenous peroxidase was blocked by incubating the tissue in TBS plus 0.3% hydrogen peroxide for 10 min. Normal goat serum (3%) in TBS with 0.25% Triton X (TBS-X) was used to block nonspecific staining for all antibodies. Slices were then incubated at 4 °C overnight with the primary antibodies (Table 2). For colorimetric immunohistochemistry, antibody binding was detected by incubating sections with biotinylated secondary antibodies (Table 2) in TBS-X. Colorization was achieved using the VECTASTAIN Elite avidin–biotin complex (ABC)-HRP kit solution (Vector Laboratories, Burlingame, CA) followed by the application of 3–3′-diaminobenzidine (3–3′-DAB, Sigma-Aldrich). Sections were mounted on glass slides in TBS-X, dried and dehydrated in 50%, 70%, 95% and twice with 100% ethanol followed by Xylene (Sigma-Aldrich) before coverslipping with Dibutylphthalate Polystyrene Xylene (DPX, Sigma-Aldrich). For fluorescent immunohistochemistry, antibody binding was detected by incubating sections with Alexa fluorescence secondary antibody (Table 2) for 2 h. Sections were mounted on glass slides in TBS-X, dried, and coverslipped with mounting medium for fluorescence with DAPI (Vector Laboratories).

We followed a published protocol for T cell identification [9]. Antigen retrieval using 10 mM sodium citrate was performed for 20 min in a 90 °C water bath followed by 30 min at RT in distilled water. After washing with TBS, slides were incubated with 1% hydrogen peroxide for 5 min, permeabilized with 0.5% Triton-X, and blocked with 10% donkey serum for 1 h in a humidified chamber. Rabbit anti-CD3 antibody (Abcam, Cambridge, UK) was incubated overnight in a humidified chamber at 4 °C. After washing again with TBS, biotinylated secondary antibody (Table 2) was incubated for 1 h in a humidified chamber. After washing with TBS, samples were incubated for 1 h with ABC-HRP kit solution and then developed using 3–3′-DAB for 30 min. Sections were mounted on glass slides in TBS-X, dried, and dehydrated as described above.

#### Cresyl violet-staining

Cresyl violet staining was used for the detection of Nissl bodies in the cytoplasm of neurons on PFA sections in order to measure lesion and hippocampal volume. After 3 washes in TBS, tissue was mounted on charged slides and dried overnight. The following day, slides were put in a cylinder glass holder and incubated in FD cresyl violet solution (FD Neurothecnologies, Inc, Colombia, MD) for 10 min. The remaining cresyl violet solution was rinsed away with water for 20 min. Then, the slides were dried and dehydrated in 95% ethanol (10 min), twice in 100% ethanol placed in xylene (8 min) before being coverslipped with DPX.

#### Measurement of cytokines and chemokines in brain supernatant

We used a modified protocol adapted from methods previously described for protein extraction [14]. The ipsilateral hippocampus was weighted and homogenized with a PowerGen model 125 homogeneizer (Fisher Scientific, Hampton, NH) in a 5 × volume of extraction buffer [20 Mm TrisHCl, 0.15 M NaCl, 2 mM EDTA, and Protease Inhibitor Cocktail (Sigma-Aldrich)]. Samples were centrifuged (3000 rpm) for 10 min at 4 °C. Then, the supernatant was removed and centrifuged a second time (13700 rpm for 40 min at 4 °C) to eliminate any remaining debris. For all samples, protein concentration was quantified with a BCA Protein Assay Kit (Pierce Biotechnology, Rockford, IL). Measurement of cytokines and chemokines in brain supernatant was performed using a mouse cytokine array (R&D systems, Minneapolis, MN), following the manufacturer’s instructions.

#### Reverse transcription-quantitative polymerase chain reaction

We used a modified protocol adapted from methods previously described for RNA extraction [43]. Total RNA was isolated from ipsilateral hippocampus using Trizol (Invitrogen, Carlsbad, CA) and DNase treated using TURBO DNA-*free* DNase Treatment Kit (Ambion, Austin, Tx). First-strand cDNA was synthesized using a High-Capacity cDNA Reverse Transcription kit (Applied Biosystems, Foster City, CA). Gene expression was assayed using the Power SYBR Green PCR Master Mix (Applied Biosystems) with primers listed below (Table 3) and quantified using the Step One Plus Real-Time PCR system (Applied Biosystems). Relative fold changes were calculated using the comparative threshold cycle methods (2-ΔΔCt).

**Table 3:** qRT-PCR primers

Genes	Forward primer	Reverse primer
qRT-PCR oligonucleotide		
Il-6	5′-CAGAGGATACCACTACCAACAG-3’	5′-TCTCATTTCCACCACGATTTCCC-3’
Il-10	5′-CAGGACTTTAAGGGTTAC-3’	5′-ATTTTCACAGGGGAGAATC-3’
Il-16	5′-GAGGTGTGGTGATGAGATTG-3’	5′-GCTTACGATGATGGGAACTG-3’
Il-17	5′-CAGCGATCATCCCTCAAAG-3’	5′-CGCCAAGGGAGTTAAAGAC-3’
Bdnf	5′-GAGCGTGTGTGACGTATTAG-3’	5′-CTTTGGATACCGGGACTTTC-3’
Cxcl13	5′-CATAGATCGGATTCAAGTTACGCC-3’	5′-GTAACCATTTGGCACGAGGATTC-3’
β-Actin	5′-CTGCCTGACGGCCAAGTCATCAC-3’	5′-GTCAACGTCACACTTCATGATGG-3’

#### Quantification of immunohistochemistry

The extent of tissue loss in the ipsilateral hemisphere for each animal was quantified using images of cresyl violet-stained slices acquired using an Axioscan slide scanner (Hamamatsu Corporation, Middlesex, NJ). Tissue loss in the injured hemisphere was calculated as a percentage of the tissue volume in the contralateral hemisphere as described by others [69]. Stereological analysis was performed using Stereo Investigator 2017 software (version 8.2; MBF Bioscience, Williston, VT). Assessments were made by an investigator blinded to group assignment. The optical fractionator function was used to quantify target markers per cubic mm of tissue. For quantification of hippocampal neuronal loss in the cornu ammonis (CA) 3 region, a grid size of 125 × 125 µm, a counting frame of 25 × 25 µm, and a dissector height of 15 µm with a guard zone of 3 µm were used for all quantifications. All ROIs were traced at 4X  magnification, and markers were counted at 100X  magnification. For stereological quantification of Iba1-positive cells, the optical fractionator function was used, with a grid size of 200 × 200 µm and a counting frame of 50 × 50 µm. The ROI began with the most anterior slice containing hippocampal dentate gyrus (DG) and ended with the most posterior section containing corpus callosum fibers that crossed the midline, which yielded 3 to 4 sections for analysis per animal. Gunderson’s coefficients of error were < 0.1 for all stereological quantifications.

Fluorescence images were taken on a Zeiss Axio Imager Z2 Fluorescence Microscope with ApoTome 2 optical sectioning grid imager with 20X objective. 20 µm z stacks with 1 µm interval were obtained of the ipsilateral DG. The DG of three serial dorsal hippocampal slices 300 µm apart from each mouse were counted for colocalization of BrdU with Dcx and NeuN.

#### Three-dimensional reconstruction of microglia

We followed a published protocol for microglia morphology analysis [15]. 50-μm sections were stained with Iba1 (Table 2) 4 °C overnight, followed by Alexa Fluor 488–conjugated secondary antibody (Table 2) staining for 2 h. Sections were mounted on glass slides in TBS-X, dried, and coverslipped with mounting medium for fluorescence with DAPI. Imaging was performed on a Zeiss LSM 880 confocal laser scanning microscope (Zeiss, White Plains, NY) using a 20X 0.8 NA objective. Z-stacks were done with 1.00-μm steps in z direction; 1,024 × 1,024 pixel resolution were recorded and analyzed using IMARIS software (Bitplane, Concord, MA). Three hippocampal cells from the ipsilateral CA3 striatum radiatum were reconstructed per analyzed mouse.

#### Blood–brain–barrier assessment

BBB permeability was measured using fluorescein isothiocyanate (FITC)-dextran (Sigma-Aldrich). Two minutes prior to sacrifice, mice were given 50 µl of 70-kDA FITC-dextran (100 mg/mL) via tail vein injection. Brains were quickly removed after decapitation and fixed by immersion in 4% PFA for 24 h, followed by 48 h in 30% sucrose. Brains were embedded in cryostat compound (Tissue-Tek, Torrance, CA), frozen on dry ice, and cut into 50-μm thick sections on a cryostat. Z-stack images at 4 mm steps of the ipsilateral hippocampus were obtained at 20X with a Zeiss AxioImager Z2 (Zeiss) equipped with an ApoTome 2 optical sectioning grid imager. Image analysis was performed with ImageJ as previously described [27].

#### Statistical analysis

All data were analyzed using GraphPad Prism 7 (La Jolla, CA). Data results are presented as mean ± standard error of the mean. There was no evidence for significant deviations from normal distribution (*p* > 0.05 by Shapiro–Wilk tests). Data were analyzed with student t-test or two-way analysis of variance with repeated measures, where necessary, followed by Tukey’s tests for multiple comparisons with a significance level of *p* < 0.05.

## Results

### Microbial dysbiosis prior to and during TBI induces worsened outcomes

**Fig. 1:**
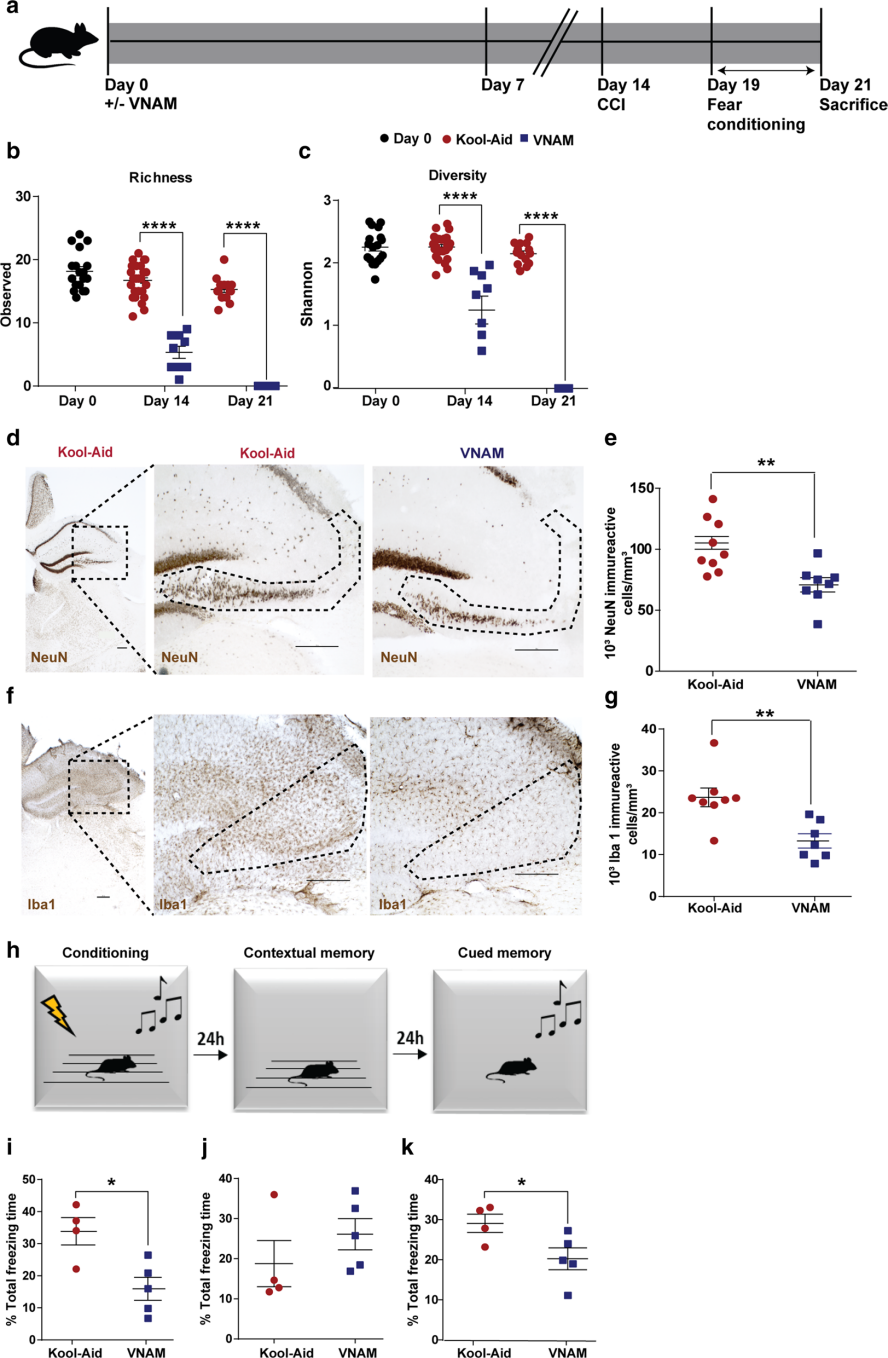
Microbial dysbiosis prior to and during TBI exacerbates neurodegeneration and fear memory alterations. **a** Experimental design. Graphs depict **b** Richness index and **c** Shannon diversity index. Genera with a frequency > 3% annotated, mean values are plotted ± SEM, unpaired t-test, **** *p* < 0.0001, n = 9–25 per group. **d** Representative image of NeuN + cells in CA3 region of injured hippocampi (indicated by the dotted line) and **e** stereological quantification. **f** Representative images of Iba1 + cells in the CA3 region of ipsilateral hippocampi (indicated by the dotted line) and **g** stereological quantification. Scale bars are 250 µm. Mean values are plotted ± SEM, unpaired t-test **p < 0.01, n = 8–9 per group. **h** Fear conditioning scheme. Quantification of % total freezing time **i** conditioning, **j** contextual memory and **k** cued memory. Mean values are plotted ± SEM, unpaired t-test, *p < 0.05, n = 4–5 per group. Abbreviations: VNAM, vancomycin, neomycin-sulfate, ampicillin and metronidazole; CCI, controlled cortical impact

Given our hypothesis that microbial dysbiosis leads to worsened outcomes after TBI, we directly investigated the effects of broad-spectrum oral antibiotics in an established murine model of TBI (Fig. 1a). We found that 2 weeks of oral antibiotics reduced the richness (Fig. 1b) and diversity (Fig. 1c) of the gut microbiota, with robust depletion of multiple genera including *Lactobacillus, Clostridium,* and *Bacteroides* (Additional file 1: Supplementary Fig. 1b). One week after injury at 3 weeks of antibiotic exposure, we observed a depletion of the gut microbiota in the antibiotic-injured mice to below the limit of detection and an expansion of *Atopobium* and *Ruminococcus* in the control-injured group (Additional file 1: Supplementary Fig. 1b).

Hippocampal neurons in the CA3 region are especially vulnerable to death post-TBI [11]. To study the association between gut microbial dysbiosis and TBI histopathological outcomes, immunostaining of ipsilateral hippocampus sections in the CA3 region was performed. We observed increased neuronal loss in antibiotic-injured mice (Fig. 1d, e) despite the fact that we did not find differences in hippocampal volume (Additional file 1: Supplementary Fig. 1c, d). This finding highlights that the increased neuronal loss in the antibiotic-injured group is not the result of local extension of hippocampal lesion volume. There is prior evidence that the absence of a complex host microbiota leads to defects in microglia maturation, differentiation, and function [15, 18]. In line with these findings, our results show a significant decrease in microglia density in the antibiotic-injured mice (Fig. 1f, g).

Evidence is also emerging that alteration of gut microbiota can also influence neural development, cognition, and behavior [59]. An alteration of fear memory response has been correlated with disruptions in hippocampal-amygdala circuitry in chronic TBI associated with post-traumatic stress disorder (PTSD) [13, 34, 37], specifically hippocampal CA3 output impairment [53, 54]. In addition, germ free (GF) mice have reduced cued fear memory response associated with functional hyperactivity in the amygdala [26]. To study the influence that microbial dysbiosis after injury has on fear memory response, we employed a 3-day setting fear conditioning experimental paradigm (Fig. 1h). On day 1 (conditioning), the antibiotic-injured mice had decreased total freezing time compared to the control-injured group (Fig. 1i). On day 2 (contextual test, a hippocampal-dependent task), we did not find differences between groups (Fig. 1j). Finally, on day 3 (cued test, a hippocampal-amygdala-dependent task), we found a significant decrease in cued fear memory in mice that had received antibiotics (Fig. 1k). Collectively, these data support the hypothesis that hippocampal-amygdala-dependent fear memory performance following TBI can be influenced by gut microbial dysbiosis.

### Innate immune activation is reduced 3 days after microbial dysbiosis at the time of injury

**Fig. 2:**
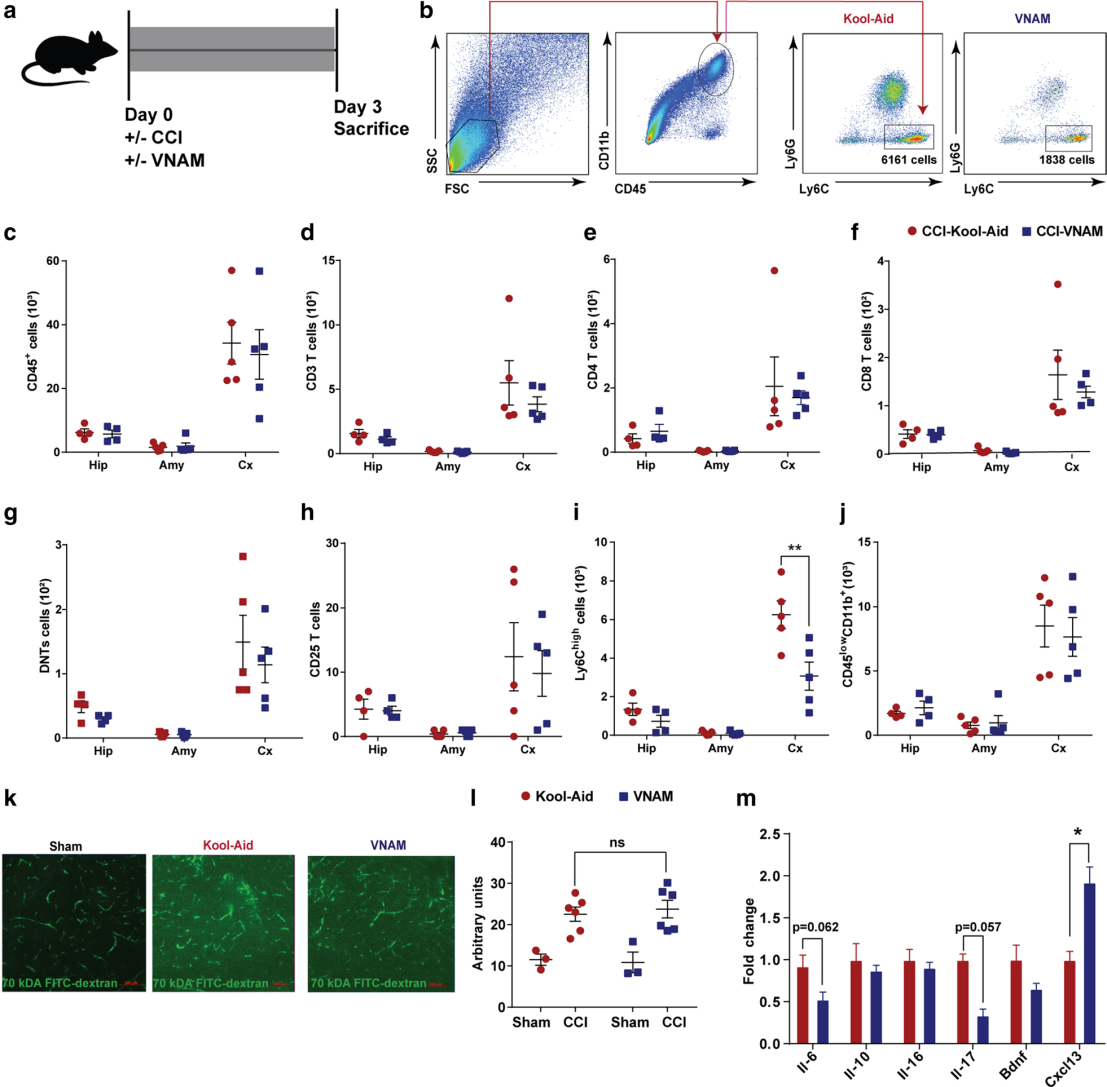
Local immune alterations in the brain 3 days after TBI and antibiotic induced gut microbial dysbiosis.** a** Experimental design. **b–j** Brain leucocyte profile characterization by flow cytometry. **b** Gating strategy of Ly6C^high^ cells. Quantification of cell absolute numbers in the hippocampi, amygdalae, and cortices for **c** leukocytes (CD45), **d** CD3 T cells (CD11b^−^CD3^+^), **e** CD4 T cells (CD11b^−^CD3^+^CD4^+^), **f** CD8 T cells (CD11b^−^CD3^+^CD8^+^), **g** DN T cells (CD11b^−^CD3^+^CD4^−^CD8^−^), **h** T reg cells (CD11b^−^CD4^+^CD25^+^), **i** monocytes (CD45^high^CD11b^+^Ly6C^+^) and **j** microglia (CD45^low^CD11b^+^). Mean values are plotted ± SEM, unpaired t test, **p < 0.01. **k** Representatives 20X images of ipsilateral hippocampi of sham, control-injured and antibiotic-injured mice. Scale bars are 100 µm. **l** Quantification of fluorescent intensity from (k), two-way ANOVA, antibiotic F_(1,14)_ = 0.015, *p* = 0.9, injury F_(1,14)_ = 29.2, *p* < 0.0001. **m** Protein cytokine and chemokine quantification from whole ipsilateral hippocampi. Mean values are plotted ± SEM, unpaired t-test, *p < 0.05. Abbreviations: VNAM, vancomycin, neomycin-sulfate, ampicillin and metronidazole; CCI, controlled cortical impact. Hip: hippocampus; Amy, amygdala; Cx, cortex

To investigate the importance of our initial findings in a clinically relevant setting, we next altered the timing of antibiotic exposure after TBI to reflect antibiotic exposure in hospitalized TBI patients. VNAM or Kool-Aid was added to the drinking water immediately after injury (Fig. 2a). We investigated the effect of microbial dysbiosis on peripheral and local immune cell populations after experimental TBI by performing flow cytometry on specific brain regions (hippocampus, cortex, and amygdala) (Fig. 2b-j) and peripheral blood (Additional file 2: Supplementary Fig. 2c-i). In the brain, we observed varying cell infiltration depending on the proximity to the injury, higher in cortex and lower in hippocampus and amygdala. In the cortex, we observed an increase of Ly6C^high^ monocytes in the control-injured group that was reduced by antibiotic exposure (Fig. 2i); however we did not find any differences in T cell infiltration between the groups (Fig. 2d-h) or the number of microglia (Fig. 2j). No differences in the immune cell populations were found in the ipsilateral hippocampus or amygdala. Analysis of peripheral blood did not reveal any immune cells changes associated with antibiotic exposure (Additional file 2: Supplementary Fig. 2c-i). It is well-characterized that BBB permeability increases after TBI [56]. However, we found that antibiotic exposure did not additionally alter BBB permeability at 3 days post-injury (Fig. 2k, l). Cytokines and chemokines are inflammatory mediators which promote inflammatory responses by increasing BBB permeability and facilitating recruitment of peripheral immune cells [14, 30]. Quantitative RT-PCR analysis results at 3 days post-injury revealed a significant increase of Cxcl13 expression in the ipsilateral hippocampus of antibiotic-injured mice compared to control-injured mice (Fig. 2m). Interestingly, Cxcl13 production in the brain is attributable to microglia [16] and increased after TBI [29]. Therefore, innate immune responses were activated 3 days after injury in the brains of control-injured mice; however, there was a significant suppression of peripheral monocyte infiltration associated with gut microbial dysbiosis.

### Peripheral immunity changes along with local immunity alteration are present 7 days after microbial dysbiosis at the time of TBI

**Fig. 3:**
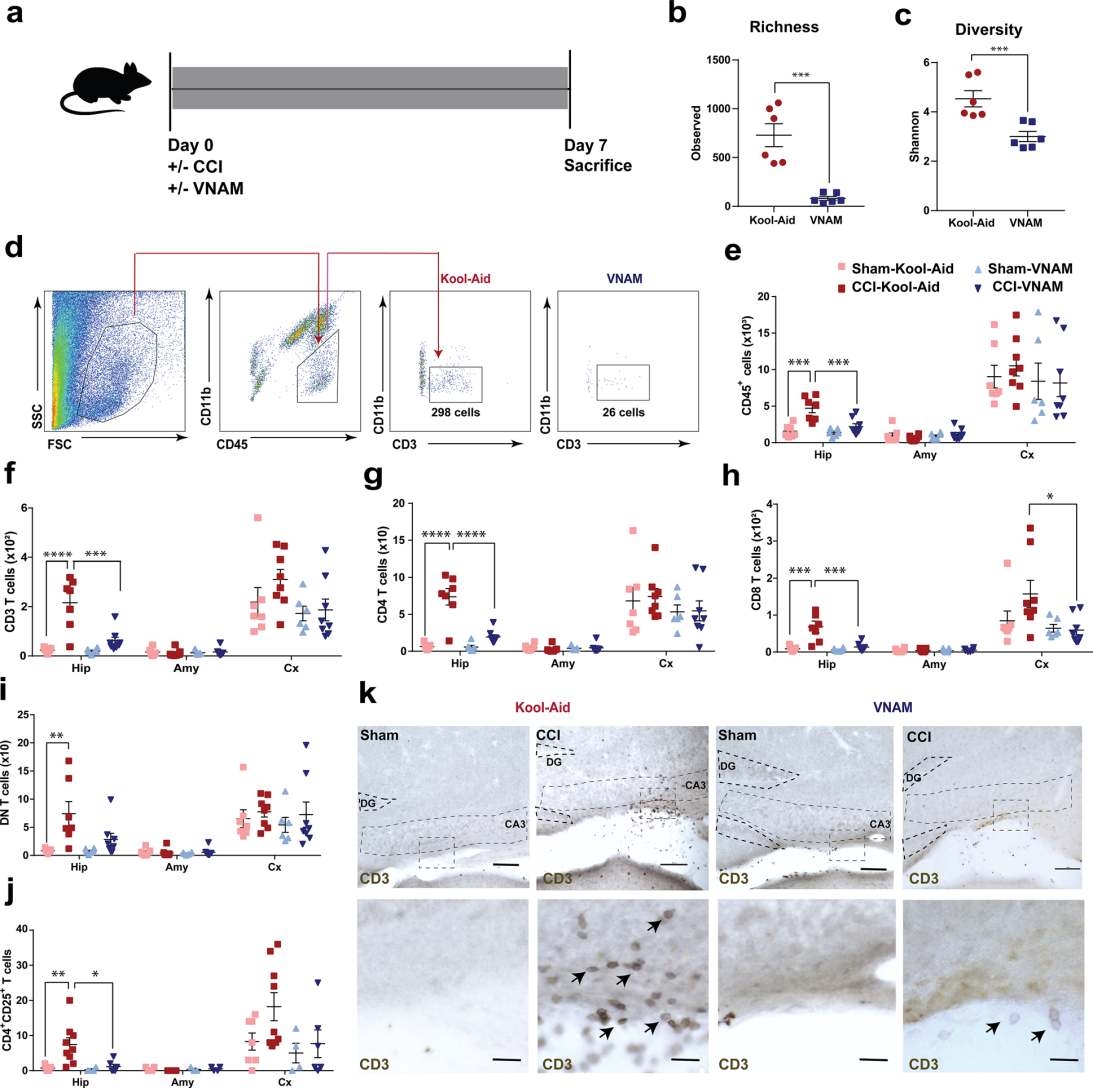
Local immune alterations in the brain 7 days after TBI and antibiotic induced gut microbial dysbiosis.** a** Experimental design. Graphs depict **b** Richness index and **c** Shannon diversity index from injured mice. Genera with a frequency > 3% annotated, mean values are plotted ± SEM, unpaired t-test, *** *p* < 0.001, n = 6 per group. **d** Gating strategy of CD3^+^ cells in the brain. **e–j** Quantification of cell absolute numbers in the hippocampi, amygdalae, and cortices. Mean values are plotted ± SEM, two-way ANOVA followed by Tukey's multiple comparisons. F statistic for antibiotic*injury presented unless otherwise noted (*p < 0.05, **p < 0.01 ***p < 0.001, ****p < 0.0001), n = 7–8 per group. **e** leukocytes (CD45), F_(1,24)_ = 7.28, *p* = 0.013; **f** CD3 T cells (CD11b^−^CD3^+^), F_(1,24)_ = 12.02, *p* = 0.002; **g** CD4 T cells (CD11b^−^CD3^+^CD4^+^), F_(1,24)_ = 19.51, *p* = 0.0002; **h** CD8 T cells (CD11b^−^CD3^+^CD8^+^), F_(1,24)_ = 9.541, *p* = 0.0053; **i** DN T cells (CD11b^−^CD3^+^CD4^−^CD8^−^), F_injury (1,24)_ = 12.11, *p* = 0.0019; and, **j** T reg cells (CD11b^−^CD4^+^CD25^+^), F_injury (1,22)_ = 6.725, *p* = 0.017, F_antibiotic (1,22)_ = 5.228, *p* = 0.032. **k** Representative 10X images (top panel, scale bars are 100 µm) and 40X (bottom panel, scale bars are 20 µm) for CD3 + staining in the ipsilateral hippocampi (as indicated by black arrows). Abbreviations: VNAM, vancomycin, neomycin-sulfate, ampicillin and metronidazole; CCI, controlled cortical impact. Hip: hippocampus; Amy, amygdala; Cx, cortex

**Fig. 4:**
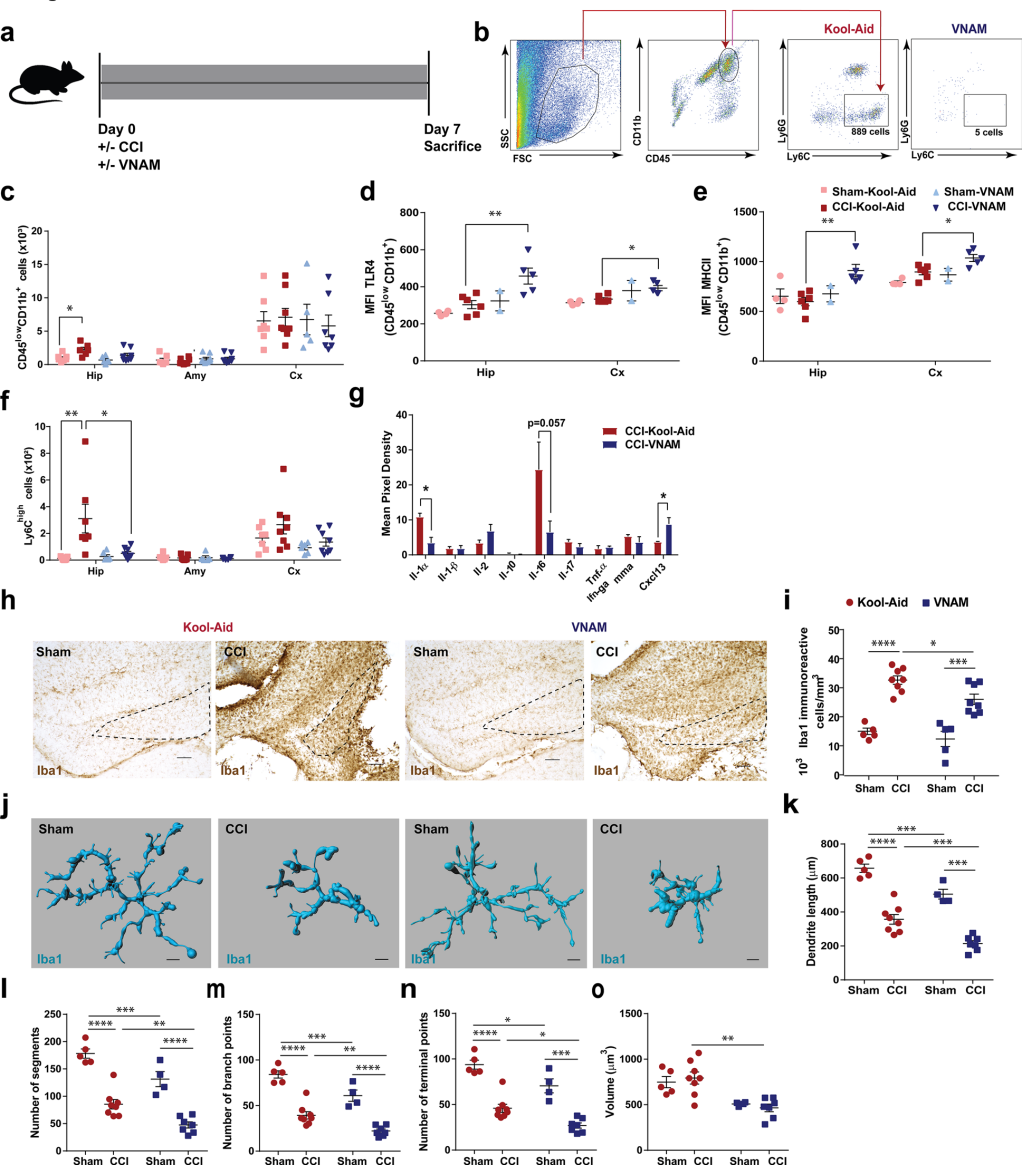
Microglia morphology changes 7 days after TBI and antibiotic induced gut microbial dysbiosis.** a** Experimental design. **b** Gating strategy of Ly6C^high^ in the hippocampus. **c–f** Quantification of absolute cell numbers in the brain. Mean values are plotted ± SEM, two-way ANOVA followed by Tukey's multiple comparisons. F statistic for antibiotic*injury presented unless otherwise noted (*p < 0.05, **p < 0.01 ***p < 0.001, ****p < 0.0001). **c** microglia (CD45^low^CD11b^+^), n = 6–8 per group, F_injury (1,23)_ = 7.45, *p* = 0.012. MFI of **d** TLR4, hippocampus F_injury (1,13)_ = 6.575, *p* = 0.024, F_antibiotic (1,13)_ = 9.9, *p* = 0.008, and cortex F_antibiotic (1,13)_ = 14.58, *p* = 0.0021; **e** MHCII, hippocampus F_antibiotic (1,13)_ = 6.32, *p* = 0.026, cortex F_injury (1,13)_ = 14.93, *p* = 0.002, F_antibiotic (1,13)_ = 9.48, *p* = 0.009, on microglia (CD45^low^CD11b^+^) gated populations. **f** monocytes (CD45^high^CD11b^+^Ly6C^+^), F_(1,24)_ = 6.24, *p* = 0.02. **g** Quantitative measurement of *Il-1α, Il-1β, Il-2, Il-10, Il-17, Tnf-α, Ifn-γ* and *Ccxl13* transcripts, unpaired t test, *p < 0.05. **h** Representative images of Iba1 + cells in the CA3 region of ipsilateral hippocampi (indicated by the dotted line) and **i** stereological quantification, F_injury (1,22)_ = 74.8, *p* < 0.0001, F_antibiotic (1,22)_ = 5.94 *p* = 0.023. Scale bars are 100 µm. **j** Representative three-dimensional reconstruction images of microglia cells. Scale bars are 15 um. **k–o** Quantification of microglia morphology. **k** Dendrite length, F_injury (1,20)_ = 124.3, *p* < 0.0001, F_antibiotic (1,20)_ = 30.9, *p* < 0.0001; **l** number of segments, F_injury (1,20)_ = 94.3, *p* < 0.0001, F_antibiotic (1,20)_ = 21.7, *p* < 0.0002; **m** number of branch points, F_injury (1,20)_ = 104.4, *p* < 0.0001, F_antibiotic (1,20)_ = 24.4, *p* < 0.0001; **n** number of terminal points, F_injury (1,20)_ = 88.0, *p* < 0.0001, F_antibiotic (1,20)_ = 18.78 *p* < 0.0004; and **o** volume, F_antibiotic (1,20)_ = 22.63 *p* < 0.0002. Abbreviations: VNAM, vancomycin, neomycin-sulfate, ampicillin and metronidazole; CCI, controlled cortical impact; MFI, median fluorescence intensity; TLR4, toll-like receptor 4; MHCII, major histocompatibility complex II

We extended our investigation of the immune system response and changes in brain pathology to 7 days post-injury (Fig. 3a). As expected, we found that 1 week of oral antibiotics reduced the richness (Fig. 3b) and diversity (Fig. 3c) of the gut microbiota in the antibiotic-injured mice compared with control-injured mice. TBI [31], stroke [4] and traumatic spinal cord injury [3] can induce a cell-mediated immune response with T cells being recruited to the brain parenchyma between 4–10 days post-injury. We found a significant increase in myeloid and lymphoid cells (CD45, Fig. 3e), CD3 T cells (Fig. 3f and k), CD4 T cells (Fig. 3g), CD8 T cells (Fig. 3h), double-negative T cells (DN, Fig. 3i) and, T-reg cells (Fig. 3j) specifically in the hippocampus of the control-injured mice compared to antibiotic-injured mice. Flow cytometry analysis of the number of microglia demonstrated a significant increase in control-injured mice compared with control-sham but no significant suppression was associated with antibiotic exposure (Fig. 4c). However, we found in the injured hippocampus and cortex an increased expression of TLR4 (Fig. 4d) and MHCII (Fig. 4e) in microglia in antibiotic-injured mice. These markers have been associated with microglial activation and cytokine production in brain injuries [1, 75]. Additionally, we found a significant decrease of monocytes (Fig. 4b and f) in the hippocampus of antibiotic-injured mice. However, we found a significant increase in monocytes in the peripheral blood of antibiotic-injured mice compared with control-injure mice (Additional file 3: Supplementary Fig. 3i). No other changes in the immune cells were found in the peripheral blood (Additional file 3: Supplementary Fig. 3c-h). To corroborate the increase in microglia number that we observed by flow cytometry in the control-injured mice, we analyzed the Iba1 + cells (marker of microglia) in the CA3 region of the hippocampus (Fig. 4h, i). We found microglia density increased in the control-injured mice and decreased with exposure to antibiotics post-injury (Fig. 4i). Within the CNS, microglial activity is governed in part by cytokines, chemokines, and neurotransmitters. We studied the expression of cytokines and chemokines in the brain at 7 days post-injury. We found a decrease in Il1-α, which is produced by activated macrophages and plays a central role in the regulation of the immune system response (Fig. 4g). Furthermore, we observed a continued increase in Cxcl13 expression in antibiotic-injured mice (Fig. 4g). These data demonstrate that modulation of gut microbiota after TBI can alter both the peripheral and resident immune cell response.

To further characterize microglial changes associated with antibiotic exposure and injury, we performed a semi-automatic quantitative morphometric three-dimensional measurements of hippocampal microglia (Fig. 4j) revealing significantly shorter dendrite length (Fig. 4k), a decreased number of segments (Fig. 4l), branch points (Fig. 4m), terminal points (Fig. 4n) and volume (Fig. 4o) in the antibiotic-injured mice compared with control-injured mice. These microglial morphological changes, along with increased pro-inflammatory markers expression support the influence of gut microbiota on microglial response after TBI.

### Gut microbial dysbiosis reduces TBI induced hippocampal neurogenesis but does not alter hippocampal neuronal loss at 7 days post-injury

**Fig. 5:**
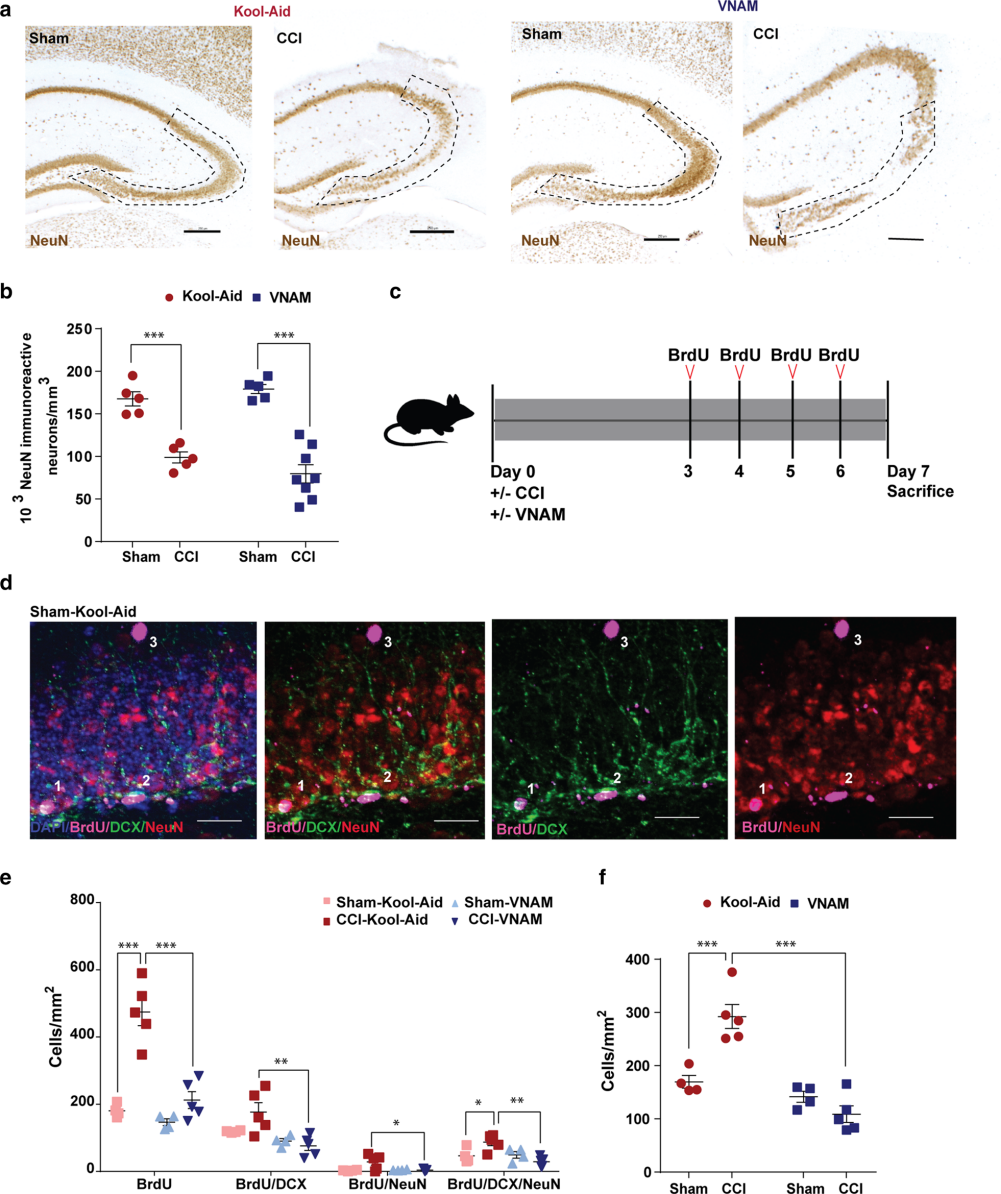
Impact of TBI and antibiotic induced gut microbial dysbiosis on neurogenesis. **a** Representative images of NeuN + cells in the ipsilateral hippocampi. Mean values are plotted ± SEM, two-way ANOVA followed by Tukey's multiple comparisons. F statistic for antibiotic*injury presented unless otherwise noted (*p < 0.05, **p < 0.01 ***p < 0.001, ****p < 0.0001). **b** Stereological quantification, F_injury (1,19)_ = 80.2, *p* < 0.0001. **c** Experimental design. **d** Representative fluoroscopy of the SGZ in the DG of the hippocampus labeled with BrdU (magenta), NeuN (red), and DCX (green) (^1^cell expressing BrdU/DCX/NeuN, ^2^cell expressing BrdU/DCX and and ^3^cell expressing BrdU only). **e** Quantification of stained cells per area per condition. **f** Summation of total neuronal lineage cells per area of hippocampi, F_(1,14)_ = 20.8, *p* < 0.0005. Abbreviations: VNAM, vancomycin, neomycin-sulfate, ampicillin and metronidazole; CCI, controlled cortical impact; SGZ, subgranular zone; DG, dentate gyrus; DCX, doublecortin; BrdU, 5-Bromo-2´-Deoxyuridine

The initiation of antibiotic exposure immediately after injury did not recapitulate our earlier findings of increased hippocampal neuronal loss in the CA3 region of the hippocampus when antibiotic-induced microbial dysbiosis was induced two weeks prior to injury (Fig. 5a, b). However, we wanted to further explore how TBI with microbiome dysbiosis could affect the cell proliferation (neurogenesis) in the DG. Studies have described neurogenesis at the subgranular zone (SGZ) of the DG in the hippocampus after TBI in rodent models [10] and humans [77] being linked to hippocampus-dependent cognitive function [66]. In addition, it has been recently found that antibiotic exposure decreased neurogenesis in the rodent hippocampus [52]. To investigate the impact of TBI with microbial dysbiosis in neurogenesis, we injected BrdU daily on post-injury days 3–6 (Fig. 5c). We stained ipsilateral hippocampus sections by immunofluorescence (Fig. 5d) and analyzed the number of BrdU-positive cells in the DG (Fig. 5e, f). Consistent with previous reports, we observed a significant increase in the number of BrdU-positive cells in the DG of control-injured mice compared with sham (Fig. 5e, f). BrdU-positive cells co-labelled with doublecortin (DCX) and/or NeuN enabled quantification of neurogenesis. The addition of antibiotics after injury was associated with decreased TBI-induced neurogenesis (Fig. 5e-f).

### Peripheral and local immunity changes persist 1 month after microbial dysbiosis at the time of TBI

**Fig. 6:**
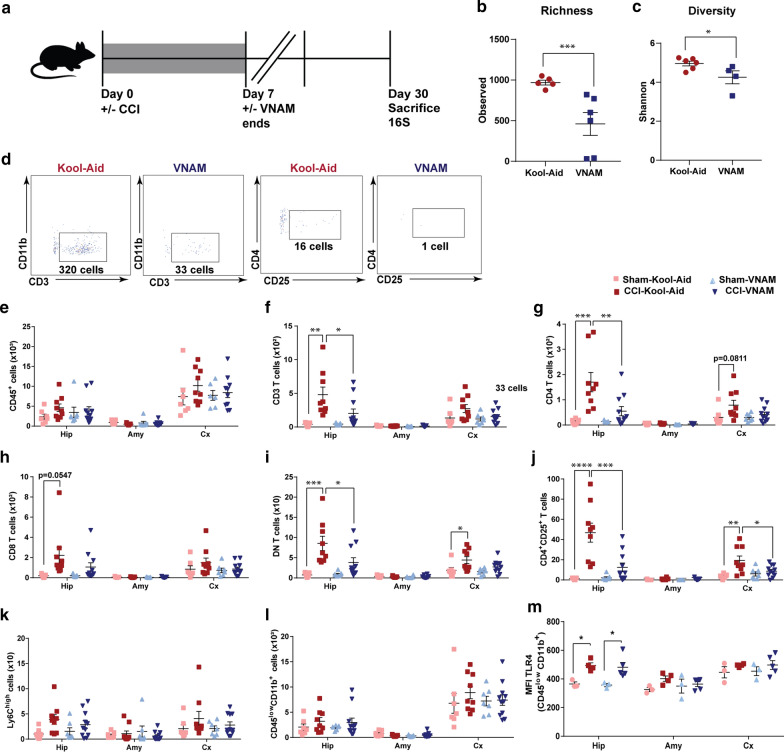
Long term alterations of local immunity after microbiota dysbiosis at the time of TBI. **a** Experimental design. Graphs depict **b** Richness index and **c** Shannon diversity index. Genera with a frequency > 3% annotated, mean values are plotted ± SEM, unpaired t-test, *** *p* < 0.001, n = 3 per group. **d** Gating strategy of CD3^+^ cells and CD25^+^ cells in the ipsilateral hippocampi. **e–l** Quantification of the cell absolute numbers in the brain. Mean values are plotted ± SEM, two-way ANOVA followed by Tukey's multiple comparisons. F statistic for antibiotic*injury presented unless otherwise noted (*p < 0.05, **p < 0.01 ***p < 0.001, ****p < 0.0001). n = 7–8 mice per group. **e** leukocytes (CD45), **f** CD3 T cells (CD11b^−^CD3^+^), F_injury (1,30)_ = 15.52, *p* = 0.0005; **g** CD4 T cells (CD11b^−^CD3^+^CD4^+^), F_(1,30)_ = 5.704, *p* = 0.023; **h** CD8 T cells (CD11b^−^CD3^+^CD8^+^), **i** DN T cells (CD11b^−^CD3^+^CD4^−^CD8^−^), F_injury (1,30)_ = 18.91, *p* = 0.0001; **j** Treg cells (CD11b^−^CD4^+^CD25^+^), hippocampus, F_(1,30)_ = 9.011, *p* = 0.0054, cortex, F_(1,30)_ = 5.443, *p* = 0.0265; **k** monocytes (CD45^high^CD11b^+^Ly6C^+^), and **l** microglia (CD45^low^CD11b^+^). **m** MFI of TLR4 in the microglia population, F_injury (1,11)_ = 19.85, *p* = 0.0010. Abbreviations: VNAM, vancomycin, neomycin-sulfate, ampicillin and metronidazole; CCI, controlled cortical impact. Hip: hippocampus; Amy, amygdala; Cx, cortex

Next, we investigated whether differences in the immune response persisted after injury (Fig. 6a). Surprisingly, at one month, we still observed differences in the richness (Fig. 6b) and diversity (Fig. 6c) of the gut microbiota between control and antibiotic-exposed mice. Flow cytometry analysis of the brain revealed a persistent reduction in lymphocytes in antibiotic-injured mice compared with control-injured mice (Fig. 6 d, f, g, i, j). Flow cytometry analysis of the blood 1 month after injury showed no changes in immune cell types (Additional file 4: Supplementary Fig. 4i-o). No changes were found in the number of monocytes (Fig. 6k) and microglia (Fig. 6l), but we detected an increase of the microglial surface marker, TLR4, in the injured mice compared with sham groups (Fig. 6m). In summary, we observed long-lasting reduction in infiltration of lymphocytes weeks after discontinuation of antibiotics.

### Gut dysbiosis is associated with alteration of fear memory response and increased hippocampal neuronal loss 3 months after TBI

**Fig. 7:**
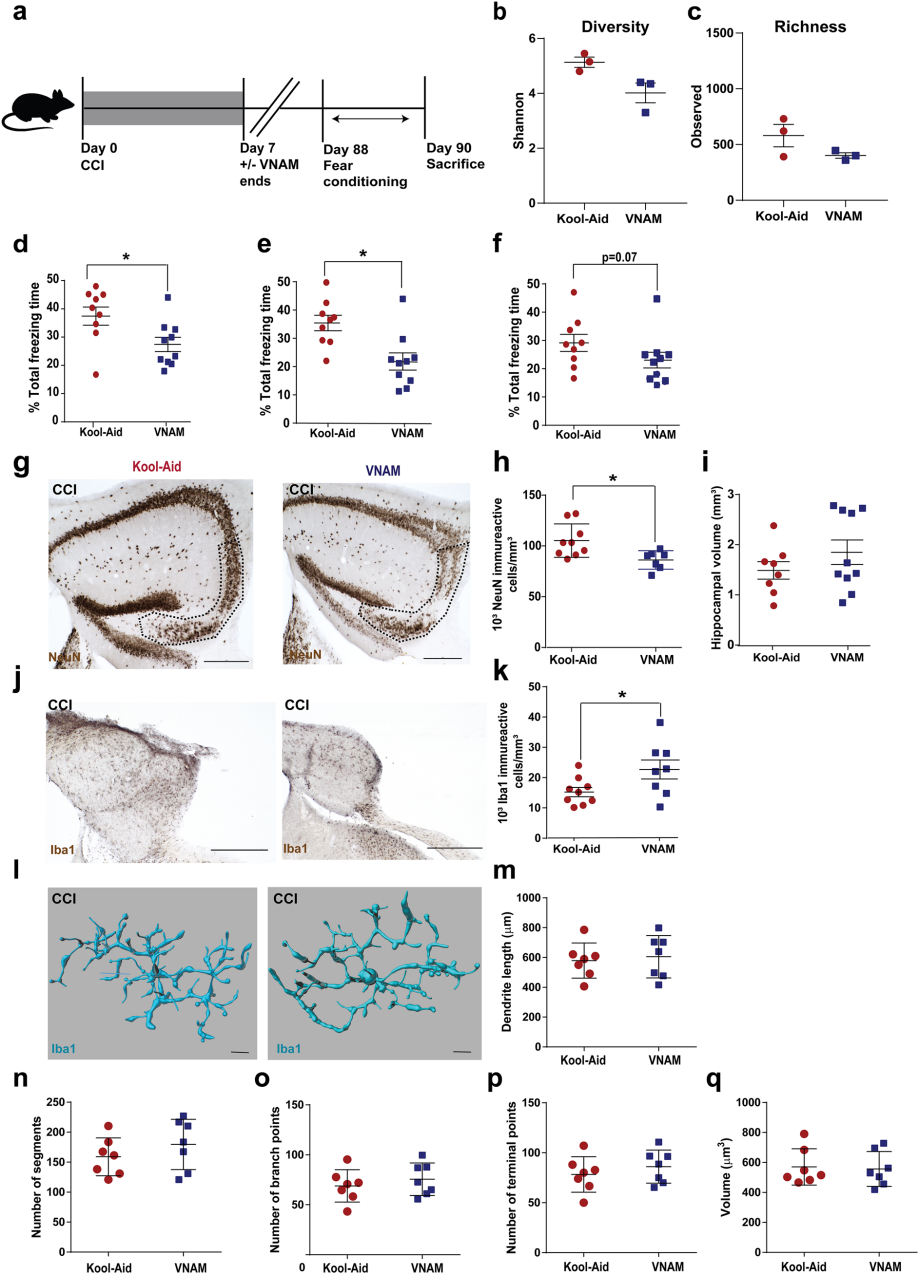
Alterations in fear memory response and subcellular populations three months after TBI in the setting of gut microbial dysbiosis.** a** Experimental design. Graphs depict **b** Richness index and **c** Shannon diversity index. Mean values are plotted ± SEM, unpaired t-test, *** *p* < 0.001, n = 3 per group. Fear conditioning 3-day paradigm and quantification of % total freezing time of **d** conditioning learning, **e** contextual memory and **f** cued memory. **g** Representative images of NeuN + cells in the pyramidal layer of CA3 (indicated by the dotted line) with **h** stereological quantification and **i** hippocampi volumes. **j** Representative images of Iba1 + cells in the injured ipsilateral hippocampi. Scale bars are 250 µm. **k** Stereological analysis of microglia density as shown in (**h**). Mean values are plotted ± SEM, unpaired t-test, *p < 0.05, n = 8–9 mice per group. **l** Representative three-dimensional reconstructive images of microglia (scale bars are 15 um). **m-q** Quantification of microglia morphology in hippocampi. **m** Dendrite length, **n** number of segments, **o** number of branch points, **p** number of terminal points and **q** volume. Abbreviations: VNAM, vancomycin, neomycin-sulfate, ampicillin and metronidazole; CCI, controlled cortical impact. Hip: hippocampus; Amy, amygdala; Cx, cortex

We observed short-term differences in fear memory performance when antibiotic-induced gut microbial dysbiosis was induced prior to injury and continued during behavioral testing. We next wanted to evaluate for long-term behavioral changes in the absence of ongoing antibiotic administration (Fig. 7a). No significant differences were found in the richness (Fig. 7b) and diversity (Fig. 7c) of the gut microbiota between the injured groups at this time point. At 1 month after injury (Additional file 4: Supplementary Fig. 4a), we did not observe any differences between injured groups in novel object recognition (Additional file 4: Supplementary Fig. 4b-e) or fear conditioning (Additional file 4: Supplementary Fig. 4f-h). Although we did not find differences in novel memory between groups (Additional file 5: Supplementary Fig. 5b-d), at 3 months after injury, we saw a significant decrease in acquisition during conditioning (Fig. 7d) and contextual fear memory (Fig. 7e) and a trend towards reduced freezing during cued fear memory (Fig. 7f) in antibiotic-injured animals compared with injured-controls. Additionally, stereological quantification of the CA3 region of the ipsilateral hippocampus demonstrated increased neuronal loss in injured animals exposed to antibiotics compared to control-injured mice (Fig. 7g, h) without differences in hippocampal volumes (Fig. 7i). Moreover, we observed a persistent increase in microglia density in the antibiotic-injured treated mice (Fig. 7j, k), but no microglia morphological changes were observed at this time point (Fig. 7l-q). Our data support that antibiotic-induced changes in the gut microbiota early after injury was associated with long-lasting microglial activation, increased neurodegeneration of the CA3 region of the hippocampus, and modulated fear memory response long after gut microbial dysbiosis had resolved.

## Discussion

Our study provides novel evidence that antibiotic-induced modulation of the gut microbiota prior to or at the time of injury leads to worsened outcomes after experimental TBI and is associated with altered microbial-host immune interactions. Mice exposed to broad-spectrum antibiotics for 2 weeks prior to and 1 week after injury developed altered gut microbiota communities and increases in neuronal loss, which triggered an alteration of fear memory. To further improve the translatability of our findings, we adjusted the timing of antibiotic exposure after TBI to reflect clinical practice in the intensive care setting. These data demonstrated that induction of microbial dysbiosis immediately after injury decreased the infiltration of monocytes and lymphocytes in the brain over time and was associated with increased hippocampal neurodegeneration, alteration of neurogenesis in the DG, and fear memory changes long after antibiotic exposure.

The depletion of commensal bacteria is considered a major health hazard of indiscriminate use of antibiotics [35]. Using a broad-spectrum antibiotic cocktail in the drinking water prior to and during TBI, we were able to dramatically alter the gut microbial composition. 16S rDNA sequencing revealed that 2 weeks of antibiotics depleted *Akkermansia, Blautia, Clostridium,* and *Lactobacillus* genera but increased the relative proportion of *Acinetobacter, Escherichia, Pelomanos,* and *Propionibacterium* genera. Consistent with other reports, bacteria could not be detected in our samples after an additional week of antibiotic treatment [57, 58]. These data highlight the bidirectional interaction between the gut and the brain that influences disease pathogenesis. Interestingly, after TBI, we found an expansion of *Atopobium* and *Ruminococcus* genera exclusively in the control-injured mice. Previous reports in mice have found acute changes (24 h post-TBI) in specific commensal microbiota. However, contrary to the findings in our control-injured mice, a decrease in the *Ruminococcus* genus was observed [71]. These differences could be attributed to facility differences, post-injury collection time, and/or the use of Kool-Aid as a vehicle in our experimental design.

Neurons in the CA3 region are especially vulnerable after TBI and susceptible to chronic neurodegeneration [11] by excitotoxic compounds and inflammatory mediators present in the extracellular space [23]. We observed increased neuronal loss in the CA3 region of the ipsilateral hippocampus in the setting of microbial dysbiosis, which may be mediated by the impairment of microbiome-immune system interactions [22, 66]. However, others have observed a neuroprotective effect of broad-spectrum antibiotics prior to brain injury in an ischemic stroke model [4] and a CCI model [65]. The opposing of these results to our data could be related to the use of alternative antibiotics influencing the changes in gut microbiota populations [4] and/or differences in injury severity, timing and region of histologic analysis [65]. Our findings were associated with changes in fear memory performance long-after the impact of antibiotics on the gut microbiome had resolved, implying that a common immune-associated mechanism underlies different aspects of hippocampal plasticity and cell renewal in the adult brain [78]. Similar to our findings, GF mice have been reported to have reduced fear memory response, although these findings were associated with reduced myelination and volume of the neocortex and prefrontal cortex underlying the gut microbiome’s impact on brain development and the inherent disadvantages of utilizing GF mice [26, 45]. However, without sham or naïve mouse groups in our behavior assessments, we cannot conclude that the impact of antibiotic-induced gut microbial dysbiosis in the setting of TBI on fear memory response was detrimental or beneficial.

TBI induces a hematogenous recruitment of monocytes and/or lymphocytes, which has traditionally been viewed as a harmful process that exacerbates brain injury [9]. However, emerging findings suggest equally important protective features [4, 19, 38]. The gut microbiota provides signals for immune system development and influences the ensuing immune responses. However, disruption of these complex and dynamic interactions can have profound consequences for host health [28]. We found that microbial dysbiosis induced an acute suppression of monocyte circulation and infiltration into the brain parenchyma with no associated changes in T cell infiltration 3 days after injury. However, 7 days after injury, TBI-induced recruitment of lymphocytes and monocytes into the hippocampus was suppressed by the alteration of gut bacteria, an effect that persisted for up to 1 month. Innate and adaptive immune cell circulation or infiltration may have been altered by multiple factors. The microbiota shapes innate immunity by promoting myeloid progenitor development and differentiation in the bone marrow [36]. Microbial dysbiosis may have disrupted the bone marrow myelopoiesis associated with TBI [32]. Additionally, the suppression of monocyte synthesis or circulation in the bloodstream might induce decreased peripheral generation and/or recruitment of T cells to the injury site and be detrimental to recovery. Recently, CD4 + T cells have been shown to play an important role in the maturation of microglia during brain development which was modulated by antibiotic-induced microbial dysbiosis [55]. Our initial characterizations of reduced T cell infiltration associated with changes in microglial morphology and neurodegeneration in antibiotic-injured mice suggest that T cell-microglia crosstalk may be an important mechanistic link of gut microbiota modulation of TBI worthy of future investigation.

Microglia, resident myeloid cells in the CNS, play an important role in life-long brain maintenance and pathology [47]. The development and maturation of microglia has been reported to be influenced by host microbiota [15]. Indeed, microglia are necessary for the proper assembly of complex neuronal networks [8]. However, in response to specific stimuli or with neuroinflammation, microglia also have the capacity to damage and kill neurons [8, 21, 25]. In our study, we detected time-dependent lasting changes in microglia after TBI that were altered by microbial dysbiosis up to 90 days post-injury. Following TBI and during antibiotic exposure, we observed reduced microglial density in the ipsilateral hippocampus. In GF mice, microglia activation molecules, such as MHCII, were no different than specific pathogen-free (SPF) mice [15]. However, after 7 days post-TBI in our model, markers of microglia activation (TLR4 and MHCII) increased in the antibiotic-injured mice. These markers were also associated with microglial complexity changes which showed shorter dendrite length and a decreased number of segments in the hippocampus compared to control groups. In contrast, Erny et al. found that antibiotic exposure in naïve SPF mice induced an immature phenotype of microglia in the cortex with a longer dendrite length and higher number of segments than the control mice. Therefore, our data indicate that these microglial changes in surface pro-inflammatory markers and in morphology may be associated with a switch of resting microglia to a pro-inflammatory phenotype; this microglial activation has been connected to chronic neuroinflammation and neurodegeneration in the injured brain [30, 76] and previously associated with microbial dysbiosis [74]. Importantly, early or late manipulation of microglial phenotype has been recently demonstrated to impact neuronal cell survival and neurogenesis after TBI [24, 73].

Delayed neurodegeneration can occur months after TBI due to a cascade of events starting with degeneration of the directly affected neurons and leading to the eventual (secondary) degeneration of neurons [63, 64] and associated with spreading of pathology [23]. Surprisingly, at 90 days, we observed exacerbation of the delayed neurodegeneration in the antibiotic-injured mice in the CA3 hippocampal region with a concomitant increase in microglia in the same region. Long-term cognitive dysfunction has been associated with TBI animal models [11, 17, 46] and demonstrated in TBI survivors [67]. Astrogliosis, microgliosis, alterations in baseline neural activity patterns, and exacerbation of axonal injury are the main influencers of aversive behavior formation [11]. In addition, modulation of gut microbiota using antibiotics reduces induced cognitive deficits [4, 52] associated with decreasing BDNF levels in the adult brain [12]. We observed an alteration of acquisition and the contextual fear memory response that was not found at an earlier time point (30 days post-injury). However, the lack of naïve and/or sham groups for these long-term assessments limits determination if these changes are enhancements or deficits in fear memory performance.

Recent studies uncovered an essential contribution of host microbiota during neurodegenerative diseases including anxiety, depression, Alzheimer’s disease (AD), Parkinson’s disease (PD), TBI, and amyotrophic lateral sclerosis [2, 49, 50]. In line with our observations, antibiotic-induced gut microbial dysbiosis in an AD mouse model (APP/PS1 transgenic mice) increased amyloid-β plaque deposition [51]. Antibiotic modulation of the gut microbiota has also been shown to beneficial effects on AD onset. The elimination of pathogenic bacteria such as Helicobacter pylori has been shown to improve cognitive and functional status parameters in AD patients [40]. Microglial Aβ clearance was enhanced in GF and antibiotic treated 5xFAD preventing neurodegeneration and cognitive deficits [50]. Additionally, in a PD mouse model gut microbiota were required for the development of motor deficits, microglia activation, and α-Synuclein pathology [60]. The protective or harmful role of antibiotic modulation of the gut microbiome in various CNS pathologies may be influenced by disease pathogenesis, temporal timing and length of antibiotic exposure, and the changes in specific microbial communities.

Together, our data demonstrates that antibiotic-induced gut microbial dysbiosis in the setting of TBI modulates the neuroinflammatory response and neurogenesis with long-term consequences on neuronal survival and fear memory. Profound alteration of the gut population early after TBI influences microglial activation as well as long-term T cell infiltration and impact neuronal degeneration and fear memory. Lack of specific bacteria populations and/or their metabolites may be critical factors influencing TBI tissue repair and long-term recovery. Further investigation of T cell-microglia interactions will be essential for understanding the mechanism underlying how early microbial dysbiosis early after TBI may have long-lasting impacts on outcomes.

## Supplementary Information

**Additional file 1**: **Supplementary Fig. 1.** No hippocampal volume changes prior to and during TBI induces microglia density changes. **a** Experimental design. **b** Fecal 16S rDNA genus frequencies on days 0, 14 and 21. **c** Representative cresyl violet stained coronal brain sections every 300 µm at 7 days after CCI from -1.6 to -2.70 Bregma points (hippocampal region indicated by dotted line). **d** Quantification of ipsilateral hippocampal volume. Scale bar is 250 µm. Abbreviations: VNAM, vancomycin, neomycin-sulfate, ampicillin and metronidazole; CCI, controlled cortical impact. (TIF 2663 KB)

**Additional file 2**: **Supplementary Fig. 2.** No immune system changes in the blood after 3 days under microbiota dysbiosis at the time of TBI.** a** Experimental design. **b** Flow cytometry gating strategy of the brain. **c-i** Peripheral immune system cell profile characterization by flow cytometry of injured mice. Quantification of the absolute number of cells in the blood for (**c**) myeloid and lymphoid cells (CD45^+^), (**d**) CD3 T cells (CD11b^-^CD3^+^), (**e**) CD4 T cells (CD11b^-^CD3^+^CD4^+^), (**f**) CD8 T cells (CD11b^-^CD3^+^CD8^+^), (**g**) DN T cells (CD11b^-^CD3^+^CD4^-^CD8^-^), (**h**) T reg cells (CD11b^-^CD4^+^CD25^+^) and (**i**) monocytes (CD45^high^CD11b^+^Ly6C^+^). Abbreviations: VNAM, vancomycin, neomycin-sulfate, ampicillin and metronidazole; CCI, controlled cortical impact. Hip: hippocampus; Amy, amygdala; Cx, cortex. (TIF 1717 KB)

**Additional file 3**: **Supplementary Fig. 3.** No immune system changes in the blood after 7 days under microbiota dysbiosis at the time of TBI.** a** Experimental design. **b** Flow cytometry gating strategy of the brain. **c-i** Peripheral immune system cell profile characterization by flow cytometry of injured mice. Quantification of the absolute number of cells in the blood for (**c**) myeloid and lymphoid cells (CD45^+^), (**d**) CD3 T cells (CD11b^-^CD3^+^), (**e**) CD4 T cells (CD11b^-^CD3^+^CD4^+^), (**f**) CD8 T cells (CD11b^-^CD3^+^CD8^+^), (**g**) DN T cells (CD11b^-^CD3^+^CD4^-^CD8^-^), (**h**) T reg cells (CD11b^-^CD4^+^CD25^+^) and, (**i**) monocytes (CD45^high^CD11b^+^Ly6C^+^). Abbreviations: VNAM, vancomycin, neomycin-sulfate, ampicillin and metronidazole; CCI, controlled cortical impact. Hip: hippocampus; Amy, amygdala; Cx, cortex. (TIF 1835 KB)

**Additional file 4**: **Supplementary Fig. 4.** No changes in behavior and peripheral immunity system one month after microbiota dysbiosis at the time of TBI.** a** Experimental design. **b** Novel object recognition paradigm. On day 1 quantification of (**c**) time in the center, (**d**) total distance. On day 3 quantification of (**e**) discrimination index. Fear conditioning 3-day paradigm and quantification of % total freezing time of (**f**) conditioning, (**g**) contextual memory and (**h**) cued memory. **i-o** Peripheral immune system cell profile characterization by flow cytometry of injured mice. Quantification of the absolute number of cells in the blood for (**i**) myeloid and lymphoid cells (CD45^+^), (**j**) CD3 T cells (CD11b^-^CD3^+^), (**k**) CD4 T cells (CD11b^-^CD3^+^CD4^+^), (**l**) CD8 T cells (CD11b^-^CD3^+^CD8^+^), (**m**) DN T cells (CD11b^-^CD3^+^CD4^-^CD8^-^), (**n**) T reg cells (CD11b^-^CD4^+^CD25^+^) and (**o**) monocytes (CD45^high^CD11b^+^Ly6C^+^). Abbreviations: VNAM, vancomycin, neomycin-sulfate, ampicillin and metronidazole; CCI, controlled cortical impact. Hip: hippocampus; Amy, amygdala; Cx, cortex. (TIF 1444 KB)

**Additional file 5**: **Supplementary Fig. 5.**. No differences in novel object recognition 3 months after microbiota dysbiosis at the time of TBI.** a** Experimental design. Novel object recognition quantification of on day 1 (**b**) time in the center and (**c**) total distance and, on day 3 (**d**) discrimination index. Abbreviations: VNAM, vancomycin, neomycin-sulfate, ampicillin and metronidazole; CCI, controlled cortical impact. (TIF 588 KB)

## Availability of data and material

The datasets used and/or analyzed during the current study are available from the corresponding author on reasonable request.
