# Supplementary figures and images for: Gut microbial dysbiosis after traumatic brain injury modulates the immune response and impairs neurogenesis
Source: Acta Neuropathol Commun. 2021 Mar 10;9:40. doi: 10.1186/s40478-021-01137-2 (PMC7944629; doi:10.1186/s40478-021-01137-2)

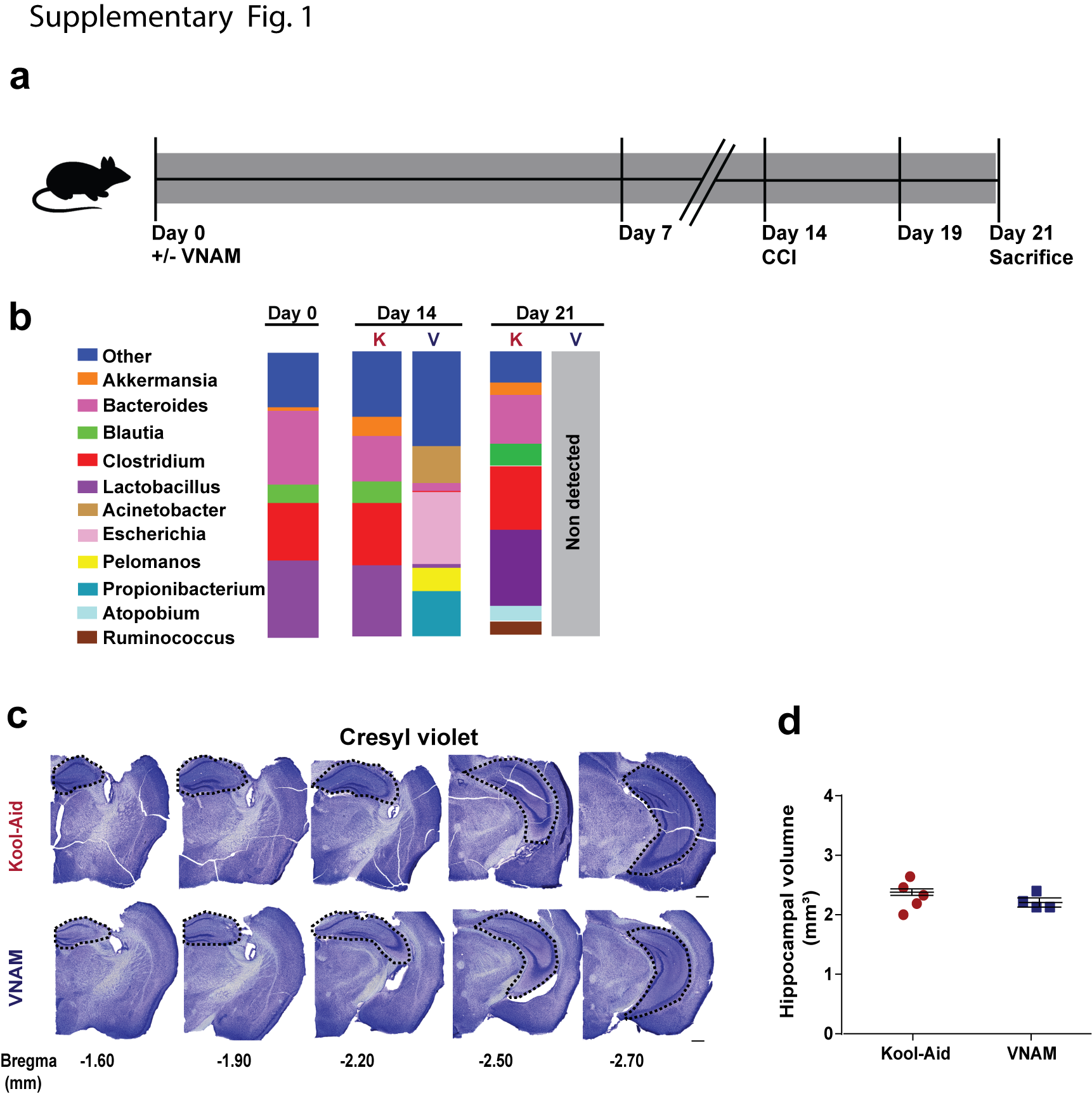

Supplement: Supplementary file 1 — Additional file 1: Supplementary Fig. 1. No hippocampal volume changes prior to and during TBI induces microglia density changes. a Experimental design. b Fecal 16S rDNA genus frequencies on days 0, 14 and 21. c Representative cresyl violet stained coronal brain sections every 300 µm at 7 days after CCI from -1.6 to -2.70 Bregma points (hippocampal region indicated by dotted line). d Quantification of ipsilateral hippocampal volume. Scale bar is 250 µm. Abbreviations: VNAM, vancomycin, neomycin-sulfate, ampicillin and metronidazole; CCI, controlled cortical impact. (TIF 2663 KB) [file 40478_2021_1137_MOESM1_ESM.tif]

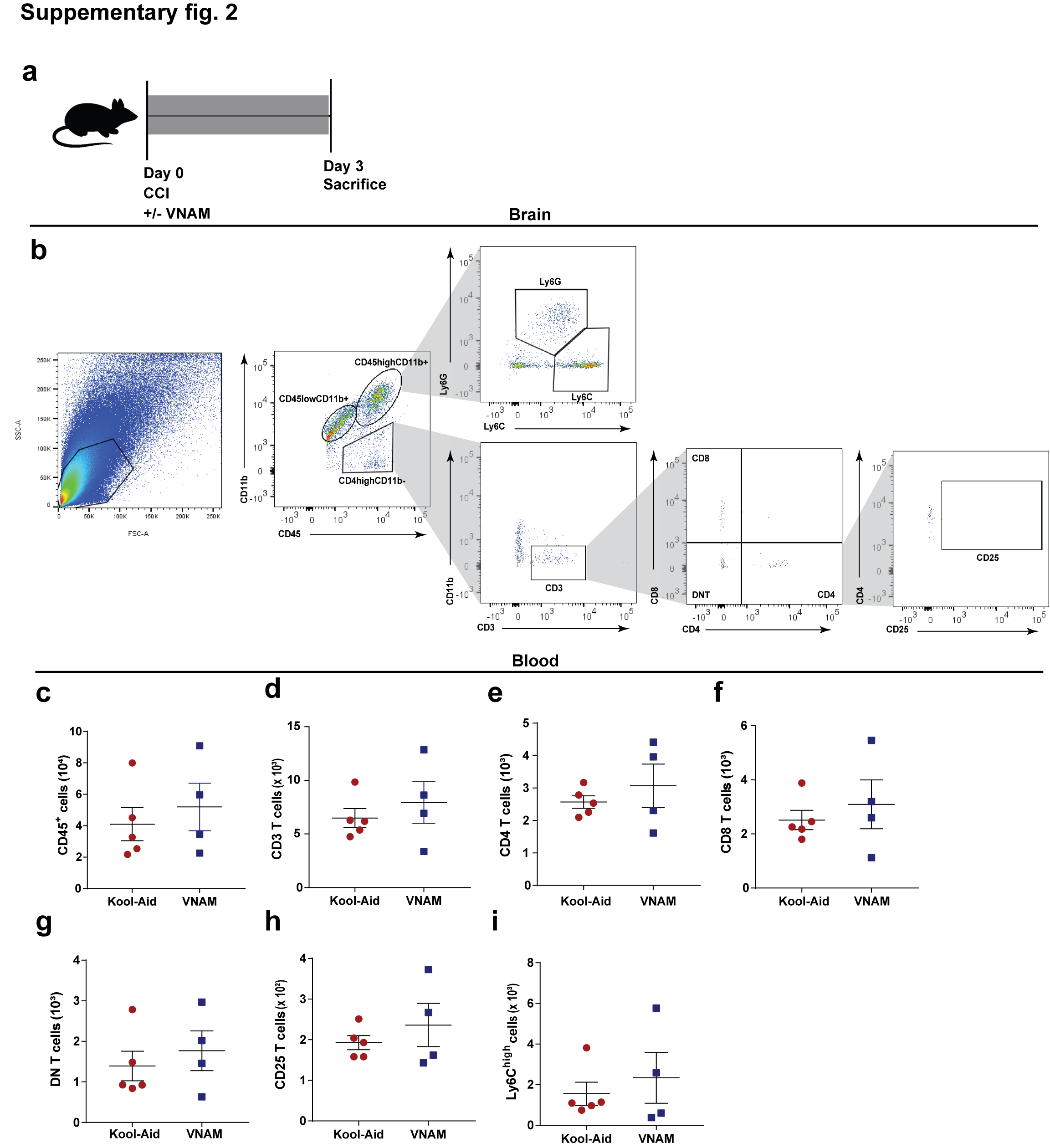

Supplement: Supplementary file 2 — Additional file 2: Supplementary Fig. 2. No immune system changes in the blood after 3 days under microbiota dysbiosis at the time of TBI. a Experimental design. b Flow cytometry gating strategy of the brain. c-i Peripheral immune system cell profile characterization by flow cytometry of injured mice. Quantification of the absolute number of cells in the blood for (c) myeloid and lymphoid cells (CD45+), (d) CD3 T cells (CD11b-CD3+), (e) CD4 T cells (CD11b-CD3+CD4+), (f) CD8 T cells (CD11b-CD3+CD8+), (g) DN T cells (CD11b-CD3+CD4-CD8-), (h) T reg cells (CD11b-CD4+CD25+) and (i) monocytes (CD45highCD11b+Ly6C+). Abbreviations: VNAM, vancomycin, neomycin-sulfate, ampicillin and metronidazole; CCI, controlled cortical impact. Hip: hippocampus; Amy, amygdala; Cx, cortex. (TIF 1717 KB) [file 40478_2021_1137_MOESM2_ESM.tif]

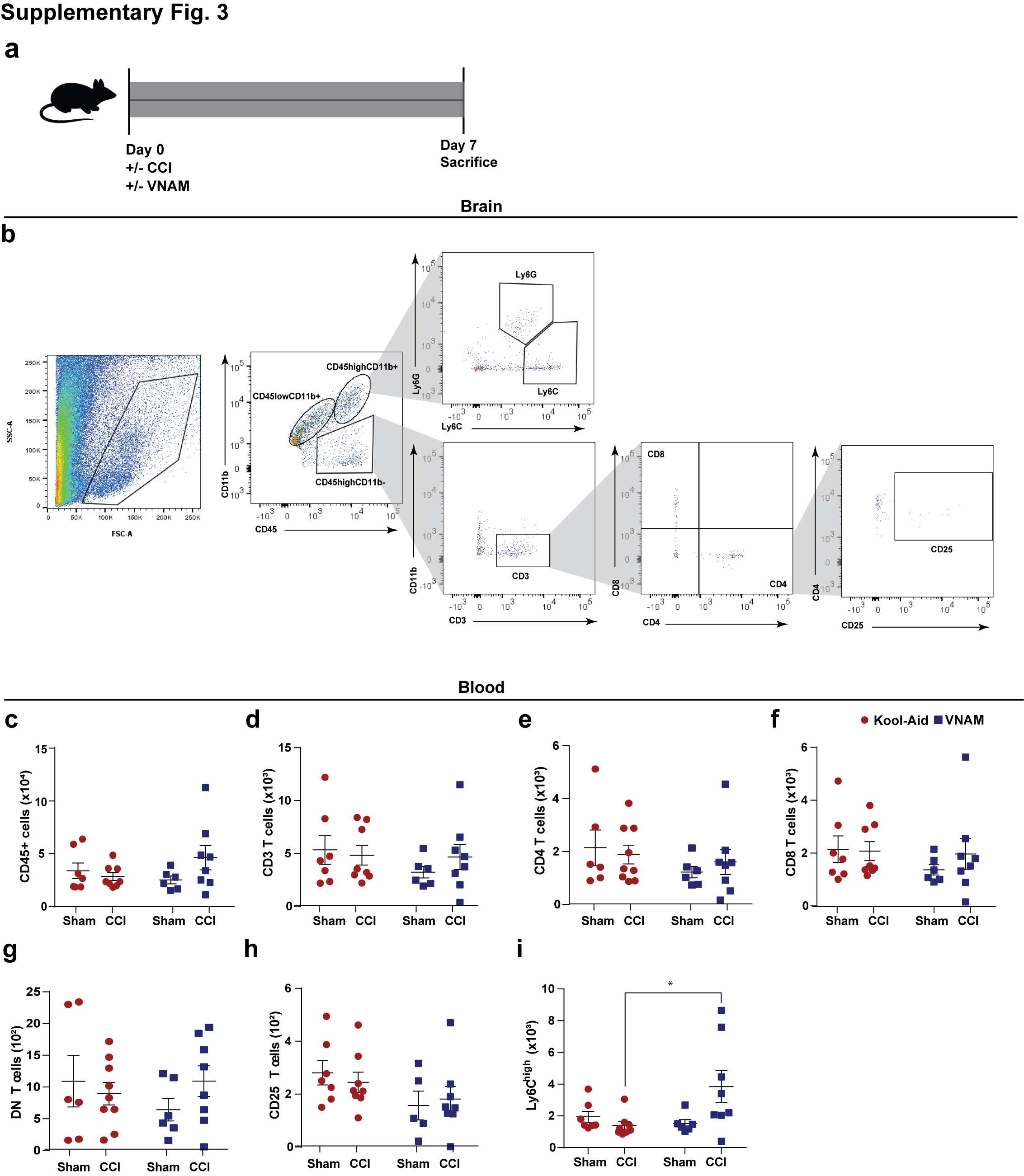

Supplement: Supplementary file 3 — Additional file 3: Supplementary Fig. 3. No immune system changes in the blood after 7 days under microbiota dysbiosis at the time of TBI. a Experimental design. b Flow cytometry gating strategy of the brain. c-i Peripheral immune system cell profile characterization by flow cytometry of injured mice. Quantification of the absolute number of cells in the blood for (c) myeloid and lymphoid cells (CD45+), (d) CD3 T cells (CD11b-CD3+), (e) CD4 T cells (CD11b-CD3+CD4+), (f) CD8 T cells (CD11b-CD3+CD8+), (g) DN T cells (CD11b-CD3+CD4-CD8-), (h) T reg cells (CD11b-CD4+CD25+) and, (i) monocytes (CD45highCD11b+Ly6C+). Abbreviations: VNAM, vancomycin, neomycin-sulfate, ampicillin and metronidazole; CCI, controlled cortical impact. Hip: hippocampus; Amy, amygdala; Cx, cortex. (TIF 1835 KB) [file 40478_2021_1137_MOESM3_ESM.tif]

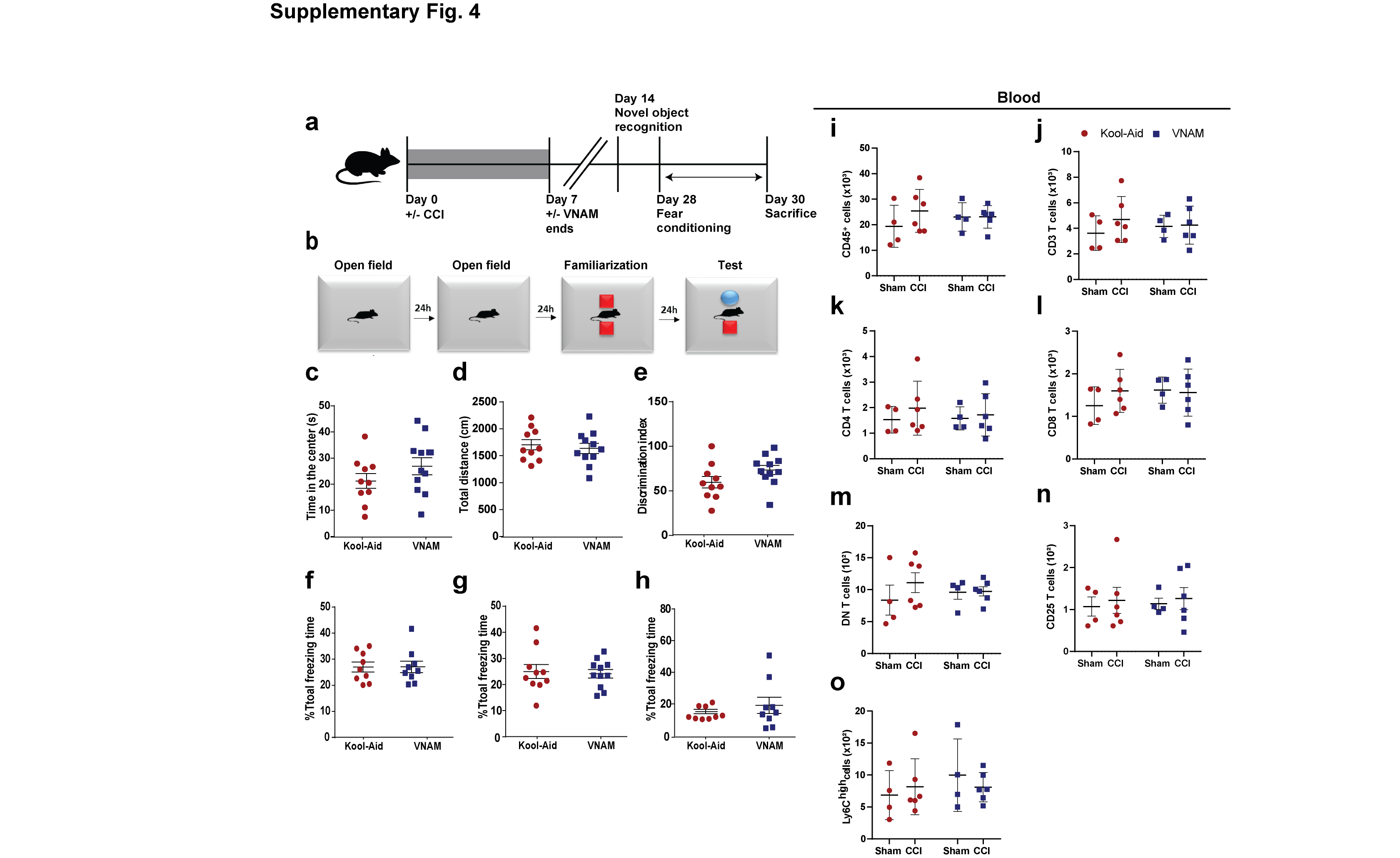

Supplement: Supplementary file 4 — Additional file 4: Supplementary Fig. 4. No changes in behavior and peripheral immunity system one month after microbiota dysbiosis at the time of TBI. a Experimental design. b Novel object recognition paradigm. On day 1 quantification of (c) time in the center, (d) total distance. On day 3 quantification of (e) discrimination index. Fear conditioning 3-day paradigm and quantification of % total freezing time of (f) conditioning, (g) contextual memory and (h) cued memory. i-o Peripheral immune system cell profile characterization by flow cytometry of injured mice. Quantification of the absolute number of cells in the blood for (i) myeloid and lymphoid cells (CD45+), (j) CD3 T cells (CD11b-CD3+), (k) CD4 T cells (CD11b-CD3+CD4+), (l) CD8 T cells (CD11b-CD3+CD8+), (m) DN T cells (CD11b-CD3+CD4-CD8-), (n) T reg cells (CD11b-CD4+CD25+) and (o) monocytes (CD45highCD11b+Ly6C+). Abbreviations: VNAM, vancomycin, neomycin-sulfate, ampicillin and metronidazole; CCI, controlled cortical impact. Hip: hippocampus; Amy, amygdala; Cx, cortex. (TIF 1444 KB) [file 40478_2021_1137_MOESM4_ESM.tif]

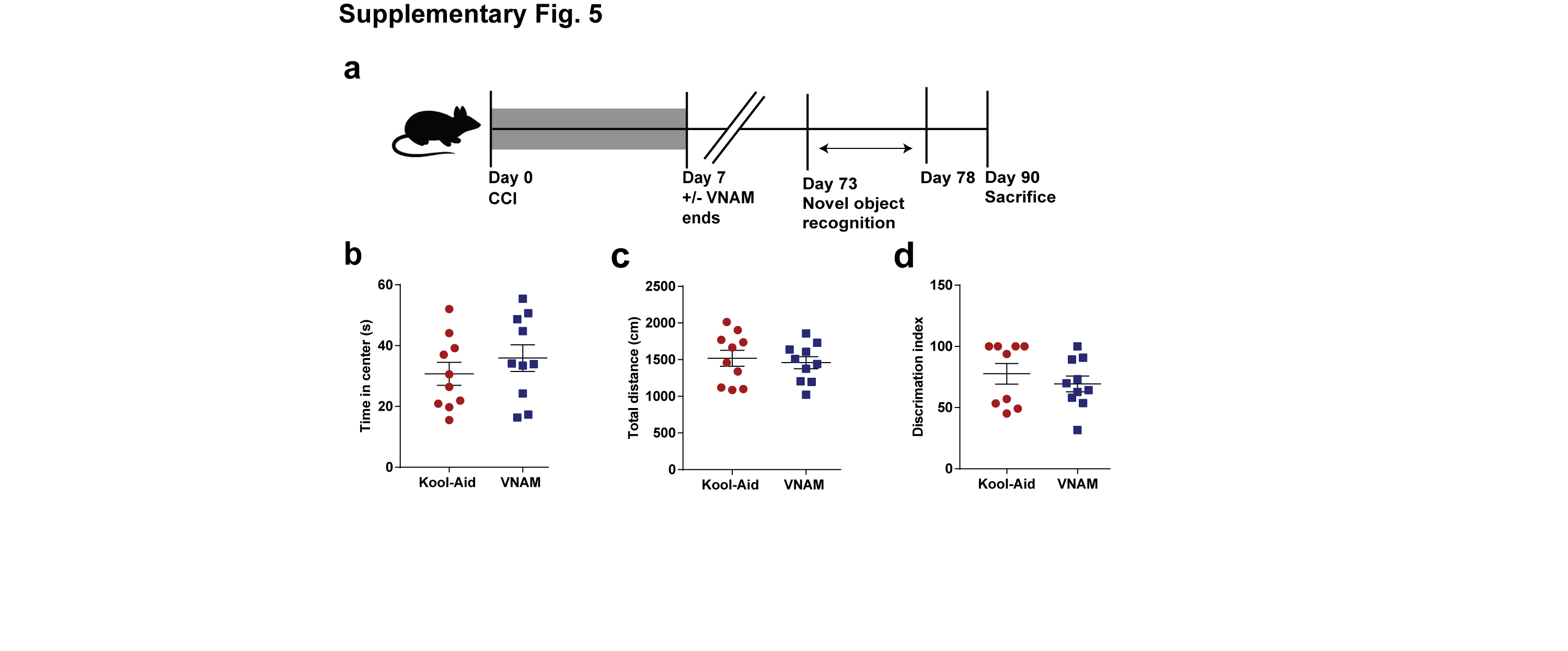

Supplement: Supplementary file 5 — Additional file 5: Supplementary Fig. 5.. No differences in novel object recognition 3 months after microbiota dysbiosis at the time of TBI. a Experimental design. Novel object recognition quantification of on day 1 (b) time in the center and (c) total distance and, on day 3 (d) discrimination index. Abbreviations: VNAM, vancomycin, neomycin-sulfate, ampicillin and metronidazole; CCI, controlled cortical impact. (TIF 588 KB) [file 40478_2021_1137_MOESM5_ESM.tif]
